# Investigation of benzylisoquinoline alkaloid biosynthetic pathway and its transcriptional regulation in lotus

**DOI:** 10.1038/s41438-018-0035-0

**Published:** 2018-06-01

**Authors:** Xianbao Deng, Li Zhao, Ting Fang, Yaqian Xiong, Collins Ogutu, Dong Yang, Sornkanok Vimolmangkang, Yanling Liu, Yuepeng Han

**Affiliations:** 1 Key Laboratory of Plant Germplasm Enhancement and Specialty Agriculture, Wuhan Botanical Garden of the Chinese Academy of Sciences, Wuhan, 430074 China; 2 Graduate University of Chinese Academy of Sciences, 19A Yuquanlu, Beijing, 100049 China; 3 Sino-African Joint Research Center, Chinese Academy of Sciences, Wuhan, 430074 China; 4 Department of Biochemistry and Microbiology, Faculty of Pharmaceutical Sciences, Chulalongkorn University, Bangkok, 10330 Thailand

## Abstract

Lotus predominantly accumulates benzylisoquinoline alkaloids (BIAs), but their biosynthesis and regulation remain unclear. Here, we investigated structural and regulatory genes involved in BIA accumulation in lotus. Two clustered *CYP80* genes were identified to be responsible for the biosynthesis of bis-BIAs and aporphine-type BIAs, respectively, and their tissue-specific expression causes divergence in alkaloid component between leaf and embryo. In contrast with the common (*S*)-reticuline precursor for most BIAs, aporphine alkaloids in lotus leaf may result from the (*S*)-*N*-methylcoclaurine precursor. Structural diversity of BIA alkaloids in the leaf is attributed to enzymatic modifications, including intramolecular C–C phenol coupling on ring A and methylation and demethylation at certain positions. Additionally, most BIA biosynthetic pathway genes show higher levels of expression in the leaf of high-BIA cultivar compared with low-BIA cultivar, suggesting transcriptional regulation of BIA accumulation in lotus. Five transcription factors, including three MYBs, one ethylene-responsive factor, and one basic helix–loop–helix (bHLH), were identified to be candidate regulators of BIA biosynthesis in lotus. Our study reveals a BIA biosynthetic pathway and its transcriptional regulation in lotus, which will enable a deeper understanding of BIA biosynthesis in plants.

## Synthesis of medicinal compounds in lotus

Researchers at the Chinese Academy of Sciences have discovered the genes in lotus responsible for the synthesis of benzylisoquinole alkaloids (BIAs), a diverse set of pharmacologically significant plant metabolites. By comparing gene expression in two lotus varieties, a high- and low-BIA cultivar, they identified the genes that were active during BIA biosynthesis as well as five transcription factors which regulate the process. One of the biosynthesis genes is expressed more strongly in the embryo and another in the leaves, leading to the accumulation of different types of BIAs in these tissues. Although many of these genes are related to known BIA synthesis genes in other species, identifying the lotus equivalents not only reveals the particulars of the process in this species but also expands our understanding of BIA synthesis in general.

## Introduction

Lotus (*Nelumbo nucifera*) belongs to Nelumbonaceae, a family of basal eudicots that is composed of only one genus, *Nelumbo*. As an aquatic perennial, lotus is widely grown in Asian countries both as an ornamental and for its edible rhizome and seed. In addition, all parts of the lotus plant have been used in traditional medicine to treat diseases such as cough, fever, hematemesis, hepatopathy, small pox, dysentery, and cholera^1^. Recently, various bioactive compounds in lotus have been identified and their significant pharmacological effects have been testified, including anti-oxidant^2^, anti-obesity^3^, anticancer^4^, antivirus^5^, and hepato-protection^6^. The main bioactive components in lotus are benzylisoquinoline alkaloids (BIAs), which are richly concentrated in tissues such as leaf and embryo^7,8^.

**Fig. 1:**
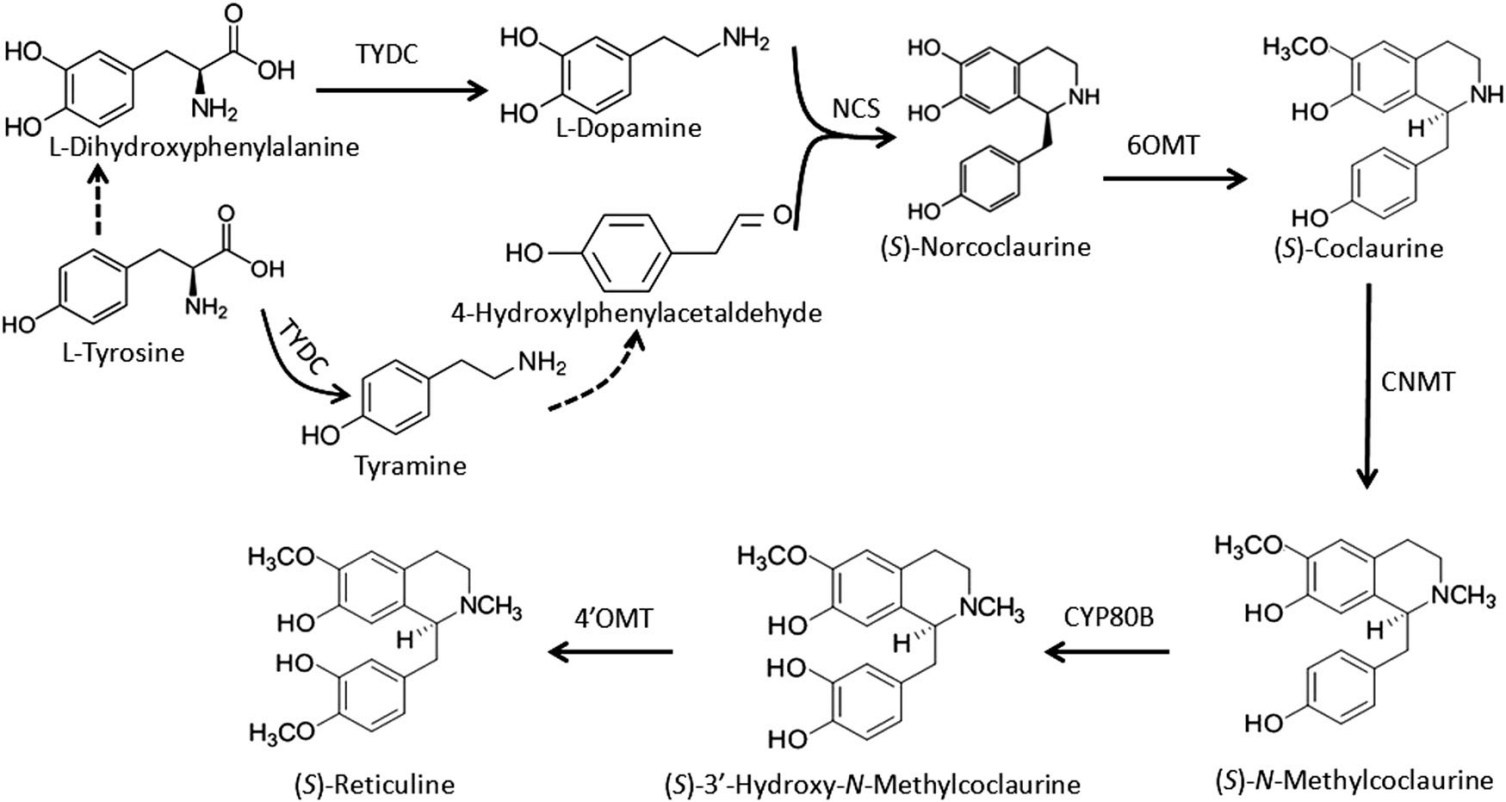
Biosynthetic pathway of the common precursor to BIAs in plants. The dash line indicates unknown reaction. TYDC tyrosine/DOPA decarboxylase, NCS norcoclaurine synthase, 6OMT norcoclaurine 6-*O*-methyltransferase, CNMT (*S*)-coclaurine *N*-methyltransferase, CYP80B, (*S*)-*N*-methylcoclaurine 3′-hydroxylase, 4′OMT 3′-hydroxy-*N*-methylcoclaurine 4′-*O*-methyltransferase

BIA alkaloids are a diverse class of nitrogen-containing plant secondary metabolites. Unlike terpenoids and phenylpropanoids that are commonly found in most higher plants, BIAs are restricted to certain plant families, including Magnoliaceae, Ranunculaceae, Papaveraceae, and Berberidaceae^9^. BIAs are structurally diverse, with approximated 2500 known BIAs, including pharmacological significant morphine and codeine (analgesic painkiller), sanguinarine and berberine (anti-microbial), noscapine (antitussive and potentially antineoplastic), and tubocuraine (muscle relaxant)^10,11^. Several plant species, such as opium poppy (*Papaver somniferum*), California poppy (*Eschscholzia californica*), Japanese goldthread (*Coptis japonica*), and yellow meadow rue (*Thalictrum flavum*), have served as models for studies of BIA biosynthesis^12–19^.

(*S*)-reticuline is well known to be the common precursor to the majority of BIAs, and its biosynthesis begins with the decarboxylation of tyrosine or l-dihydroxyphenylalanine (DOPA) to yield 4-hydroxyphenylacetaldehyde (4-HPAA) and l-dopamine, respectively, under the catalyzation of tyrosine/DOPA decarboxylase (TYDC) (Fig. 1). Subsequently, norcoclaurine synthase (NCS) catalyzes condensation of l-dopamine and 4-HPAA to form (*S*)-norcoclaurine, the first BIA scaffold in plants^20^. Four additional enzymatic steps, 6-O-methylation, N-methylation, 3′-hydoxylation and 4′-O-methylation, catalyzed by norcoclaurine 6-*O*-methyltransferase (6OMT), (*S*)-coclaurine *N*-methyltransferase (CNMT), (*S*)-*N*-methylcoclaurine 3′-hydroxylase (CYP80B), and 3′-hydroxy-*N*-methylcoclaurine 4′-*O*-methyltransferase (4′OMT), respectively, yield the central intermediate (*S*)-reticuline. Later, a variety of additional reactions, such as internal carbon–carbon, carbon–oxygen phenol coupling, and functional group substitutions, result in the formation of different types of BIAs. To date, significant efforts have been made to investigate biosynthetic pathways of BIAs, and enzymes involved in the biosynthesis of structurally diverse BIAs, such as morphine, berberine, and sanguinarine, have been nearly completely identified^11,21^. More recently, high-throughput sequencing approaches have accelerated isolation of novel BIA biosynthetic and regulatory genes in plants^22,23^.

In lotus, BIA alkaloids represent the major active components and a total of 45 BIAs have been so far isolated^7^. Our recent study revealed that two organs, laminae and embryos, contain the highest amount of BIAs, followed by petals and petioles^8^. Moreover, significant variation in BIA component has been also observed between different organs^24,25^. Overall, lotus lamina predominantly accumulates aporphine-type BIAs such as nuciferine and its derivates, whereas embryo mainly contains dimeric bis-BIAs (bis-BIAs), such as liensinine, isoliensinine, and neferine. The total concentrations of BIAs in the leaf and embryo can reach 1.41% and 2.43% on the dry weight basis, respectively^8,26^.

Lotus BIA alkaloids are well known for a variety of pharmacological effects and have been used to treat various diseases. However, little is known about the molecular mechanisms underlying the biosynthesis and regulation of BIAs in lotus. Although enzymes catalyzing the conversion of tyrosine to (*S*)-reticuline are common to all BIAs, the BIA biosynthetic pathway is flexible in plants. Both structural and regulatory genes involved in BIA biosynthesis remain largely unknown in lotus. Recently, our evaluation of lotus germplasm for alkaloid content revealed that BIA content in the leaf varies considerably among cultivars and nuciferine is the major component of alkaloids in the leaf^8^. In this study, comparative transcriptome analysis was conducted between a high-BIA cultivar Luming (LM) and a low-BIA cultivar WD40 (WD). The contents of BIAs and nuciferine in mature leaf of cv. LM are approximately 4-fold and 6-fold higher than in mature leaf of cv. WD, respectively. Our aim was to explore structural genes and transcription factors (TFs) involved in BIA biosynthesis in lotus. The results will facilitate our deeper understanding of the biosynthetic pathway of BIAs and regulatory mechanism of BIA accumulation in plants.

## Materials and methods

### Plant materials

Two lotus cultivars used in this study, LM and WD, were grown separately in a square concrete pond at Wuhan Botanical Garden of the Chinese Academy of Sciences (Wuhan, Hubei province, China). Leaf samples at different developmental stages^8^ were collected during the summer of 2016, frozen immediately in liquid nitrogen, and stored in −80 °C freezer until use.

### RNA extraction, RNA-Seq library construction, and sequencing

Leaves at two developmental stages of two cultivars were subjected to RNA-Seq library construction. For each stage, leaf samples represented a pool of five leaves, each of which was collected from different plants. Frozen leaf samples were ground into fine powder in liquid nitrogen with a mortar and pestle, and total RNA was isolated using Tiangen RNA Extraction Kit (Tiangen Biotech, Beijing, China) according to the manufacturer’s instructions. RNA integrity was evaluated using 1% agarose gel electrophoresis. Purified RNA was quantified using a Nanodrop Lite spectrophotometer (Thermo Scientific, CA, USA) and analyzed using a Bioanalyzer 2100 system (Agilent Technologies, Santa Clara, CA, USA). All samples showed a 260/280 ratio >1.8, with the RNA integrity number >8.0. Finally, RNA concentrations were measured using Quibit 2.0^®^ Flurometer (Life Technologies, Carlsbad, CA, USA) and the Qubit^TM^ RNA HS Assay Kit.

The RNA-Seq library was constructed using the NEB-Next Ultra Directional RNA Library Prep Kit for Illumina (New England Biolabs, Ipswich, MA, USA) according to the manufacturer’s instructions. Briefly, poly(A)-containing mRNAs were purified from 3 µg of total RNA using oligo (dT)-attached magnetic beads (Life Technologies, Carlsbad, CA, USA), and then fragmented using divalent cations under elevated temperature in NEB-Next First-Strand Synthesis Reaction Buffer (5×). M-MuLV reverse transcriptase (RNase H) and random hexamers were used for first-strand cDNA synthesis and the second-strand cDNA synthesis was subsequently synthesized using DNA polymerize I and RNAse H. The double-stranded cDNA was purified with AMPure XP beads (Beckman Coulter, Beverly, MA, USA), end repaired, and poly(A) end added at the 3′ end. cDNA fragments of approximately 200 bp in size were finally selected for PCR enrichment that was performed using the NEB Universal PCR Primer and Index primer. The constructed library was quantified using the Agilent Bioanalyzer 2100 system.

The clustering of the index-coded samples was performed on a cBot Cluster Generation System using TruSeq PE Cluster Kit v3-cBot-HS (Illumia) according to the manufacturer’s instructions. RNA-Seq libraries were sequenced on an Illumina Hiseq platform (Novogene, Beijing, China) and 150-bp paired-end reads were generated.

### Analysis of RNA-Seq data

The raw reads were cleaned by removing adapter sequences and low-quality regions. Clean reads were mapped to the genome sequence of the sacred lotus^27^ using TopHat v.2.0.12^28^. The number of read counts per gene locus was summarized by the HTSeq v0.6.1^29^. Gene expression levels were calculated based on fragments per kilobase of transcript per million fragments mapped. The read counts were standardized between samples by scaling the number of reads in a given library to a common value across all sequenced libraries in this study^30^.

Differentially expressed genes were identified using the DEGSeq R package (ver. 2.1.0). A false discovery rate threshold of <0.05 and a log 2 fold change of 1 were used to determine differentially expressed genes. Annotation of differentially expressed genes was performed according to the results of a Blast query against the NCBI RefSeq nucleotide database and the Swiss-Prot and UniPro protein databases, followed by the pathway annotation pipelines, including Gene ontology (GO) (http://www.geneontology.org) and Kyoto Encyclopedia of Genes and Genomes (www.genome.jp/kegg/).

### Quantitative real-time PCR

Total RNA was extracted using RNAprep Pure Plant Kit (Tiangen Biotec, Beijing, China) according to the manufacturer’s instructions. RNA samples were treated with DNase I (TaKaRa, Dalian, China) to remove any genomic DNA contamination. cDNA synthesis were conducted using cDNA Synthesis SuperMix (TransGen Biotech, Beijing, China) according to the manufacturer’s instructions. A SYBR Green-based real-time PCR assay was performed in Applied Biosystems StepOnePlus^TM^ Real-Time PCR System (Applied Biosystems), with a total volume of 25 μl reaction mixture containing 12.5 μl of 2× SYBR Green I Master Mix (Takara, Dalian, China), 0.2 μM of each primer, and 100 ng of template cDNA. The amplification program consisted of one cycle of 95 °C for 10 min, followed by 40 cycles of 95 °C for 30 s and 60 °C for 30 s. Fluorescence readings were consecutively collected during the melting process from 60 to 90 °C at a heating rate of 0.5 °C/s. An actin gene was used as a constitutive control^31^. The relative expression of genes was calculated using the 2^inu−∆∆ct^ method^32^. All analyses were performed in three biological replicates. The primer sequences are listed in Table S1.

### Lotus alkaloid extraction and quantification

Alkaloid extraction was performed according to our previously reported protocol^8^. Briefly, fresh samples were ground to fine powder in liquid nitrogen and approximately 600 mg of powder were soaked in 7 ml of extraction buffer (0.3 M HCl-methanol, 1:1, v/v). The mixture was sonicated for 30 min at room temperature and then centrifuged at 11,000 × *g* for 10 min. The supernatant was transferred to an eppendorf tube, and the pellet was extracted again with 5 ml of extraction buffer. The supernatants were combined and diluted with extraction buffer to a final volume of 15 ml. The alkaloid extracts were filtered through 0.22 µm membranes (Shanghai New Asia Purification Device Factory, Shanghai, China) and then subjected to the concentration measurement using high-performance liquid chromatography according to the protocol described by Deng et al ^8^.

### Dual luciferase reporter assay

Transient dual luciferase reporter assay was conducted in tobacco (*Nicotiana benthamiana*) according to our previously reported protocol^33^. Briefly, promoter regions upstream from the start codon of four lotus gene, *NnTYDC1* (1.8 kb), *NnNCS1* (2.2 kb), *NnCYP80G* (1.4 kb), and *Nn7OMT2* (2.1 kb), were isolated and inserted individually into the MCS of vector pGreenII 0800-LUC. Full CDS of five lotus TFs, *NnMYB6*, *NnMYB12*, *NnMYB113*, *NnbHLH1*, and *NnRAV1*, were isolated and inserted into pSAK277 vectors under the 35S promoter. Genomic DNA and RNA templates were prepared from young leaves of cv. LM. The sequences of primers used for vector construction are listed in Table S1. All constructs were transformed individually into *Agrobacterium tumefaciens* strain GV3101, and incubated at 28 °C for 2 days. The confluent bacteria were resuspended in 10 ml infiltration buffer containing 10 mM MgCl_2_, 50 μM acetosyringone, and 10 mM 2-(*N*-morpholine)-ethanesulfonic acid (pH = 5.7). Before infiltration, the bacteria were incubated at room temperature without shaking for at least 2 h. Transient transformation was conducted by mixing *Agrobacterium* GV3101 culture transformed with the reporter cassette with an equal volume *Agrobacterium* culture transformed with a cassette containing *NnMYB6*, *NnMYB12*, *NnMYB113*, *NnbHLH1*, or *NnRAV1* fused to the 35S promoter. All tests were repeated at least three times using biological replicates. Firefly luciferase (Luc) and Renilla luciferase (Ren) activity were measured 3 days after infiltration using Dual-Glo^®^ Luciferase Assay System (Promega, Madison, WI, USA) on an Infinite M200 luminometer (Tecan, Mannerdorf, Switzerland). Promoter activation ability was shown as the ratio of Luc to Ren activity.

## Results

### Dynamic alkaloid concentration in lotus leaf throughout the whole developmental stages

**Fig. 2:**
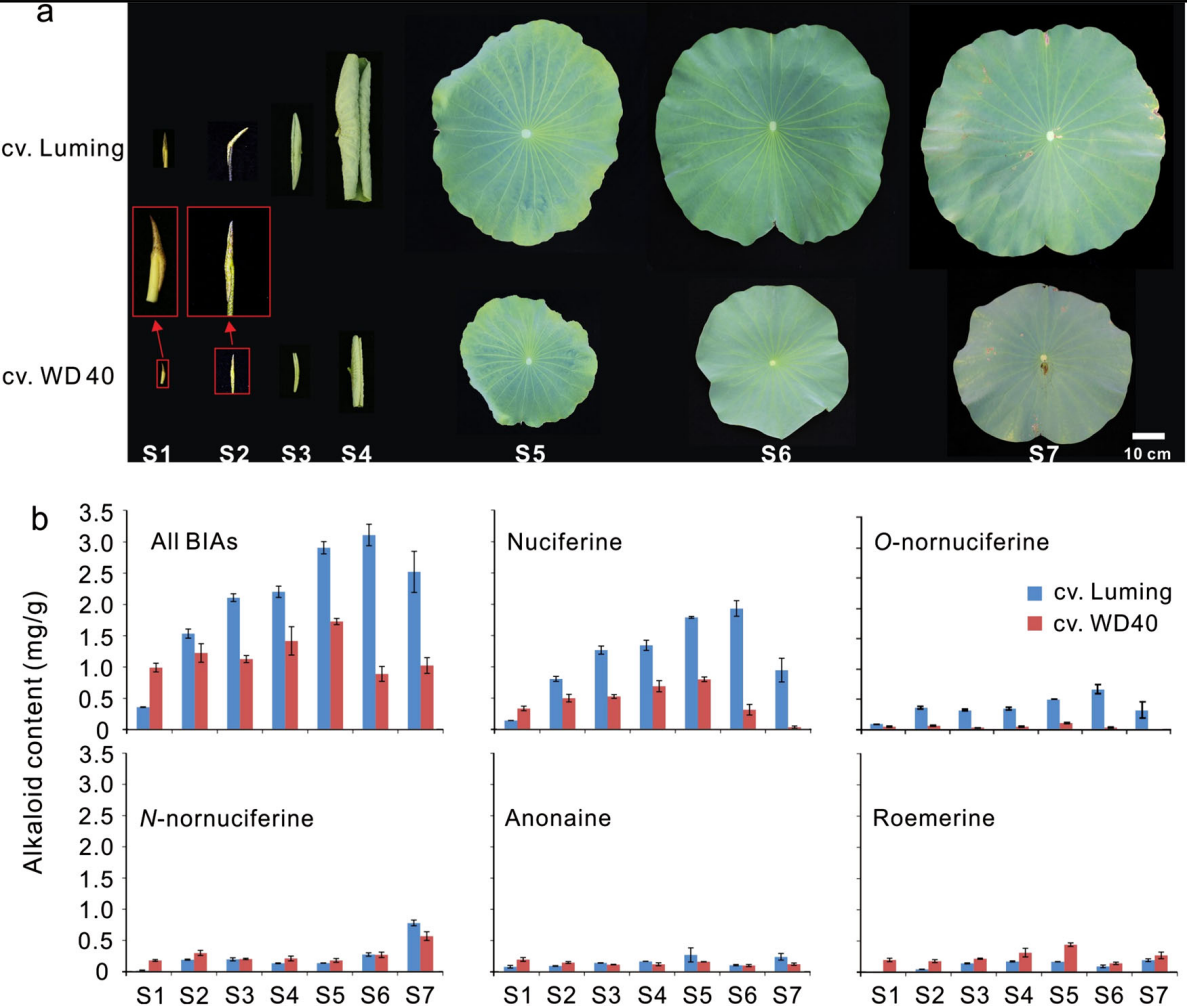
Profile of lotus alkaloids in leaves at different developmental stages. Leaves at seven developmental stages (**a**) and their corresponding alkaloid contents (**b**) of two cultivars Luming and WD40. Stage 1, leaf is under the soil and covered by sheath, with fusiform shape and pale yellow color interspersed with deep purple pots; stage 2, leaf has fusiform shape and tender green color and is out of the soil and still under the water surface; stage 3, leaf sticks out of the water surface, with fusiform shape and tender green color; stage 4, leaf is half-opened, with curly edge and tender green color; stage 5, leaf is fully opened with disk shape, with the upper epidermis being tender green color; stage 6, leaf is fully opened with disk shape, with the upper epidermis being dark green; stage 7, leaf starts to show senescence symptoms, with part of leaf edges changing color from green to yellow.

In this study, we mainly focused on BIA biosynthesis in lotus leaf as it produces a considerable amount of alkaloids. Our recent study revealed that two *N. nucifera* cultivars, LM, and WD, contain relatively high to low levels of BIAs in the leaf, respectively^8^. Overall, cv. LM with larger lamina discs accumulated higher levels of alkaloids in the leaf through all the seven developmental stages, ranging from the bud stage to the senescence stage, compared with cv. WD (Fig. 2a). Total alkaloid amount in the two cultivars increased steadily during the early stages, reached a peak at stages 5 or 6, and decreased slightly after the mature stage (Fig. 2b). In addition, two major BIA components, nuciferine and *O*-nornuciferine, showed a great difference in concentration between the two cultivars, with the highest fold change of 26.3 at stage 7 or 24.1 at stage 6, respectively. By contrast, the rest three components, *N*-nornuciferine, anonaine, and roemerine, had very low concentration in the leaf throughout the whole developmental stages, and showed a small difference between the two cultivars. Given the fact that nuciferine is the major component of BIAs in lotus leaf, these two cultivars are deemed to be suitable for comparative transcriptome study.

Our previous study shows that the *NnNCS7* gene involved in the catalyzation of the first committed step in the alkaloid biosynthetic pathway is predominantly expressed in lotus leaf and its expression level is significantly correlated with alkaloid content^34^. To determine the leaf developmental stages that are suitable for comparative transcriptome study, we investigated the expression profile of *NnNCS7* in the leaf. The *NnNCS7* gene showed similar expression profiles between cvs. LM and WD, and its expression level reached a peak in the leaf at stage 4 (Fig. S1). This suggests that the expression of the alkaloid pathway genes reached the peak earlier than did alkaloid accumulation. In addition, alkaloid content in the leaf at stage 1 was significantly lower compared with that in the leaf at stage 4 (Fig. 2b). Thus, leaf samples of cvs. LM and WD at stage 1 and stage 4 were selected and subjected to later comparative transcriptome analysis.

### Global gene expression patterns in leaves at different developmental stages

Four RNA-Seq libraries, designated LM_S1, LM_S4, WD_S1, and WD_S4, were constructed and sequenced for leaf samples of cvs. LM and WD at stage 1 (S1) and stage 4 (S4), respectively. Overall, the raw reads for each library ranged from 44.10 to 53.77 million reads, with an average of 48.20 million reads (Table 1). The raw reads were trimmed by removing adapter, empty reads, and low-quality sequences. As a result, an average of 47.14 million clean reads was generated for each library, with 7.07 Gb in size and approximately 45.6% of GC content. Approximately 75.2% of clean reads were mapped to the lotus reference genome^27^. Of the clean reads mapped, 99.2% and 0.8% were single-mapped and multiple-mapped reads, respectively.

**Table 1:** Summary of lotus leaf transcriptome data

Sample	No. of reads (million)		No. of mapped clean reads (million)			GC content of clean reads	No. of transcripts	
	Raw	Clean	Single-mapped	Multiple-mapped	Total		Total	Novel
LM_S1	53.77	52.71	40.29	0.34	40.63	44.95%	24728	1079
LM_S4	48.55	47.54	35.88	0.26	36.14	45.55%	24667	1105
WD_S1	46.39	45.45	34.06	0.31	34.37	45.95%	24782	1056
WD_S4	44.10	42.85	30.44	0.26	30.70	45.77%	24640	1063

The mapped reads were subjected to transcript discovery, and 29,489 genes were identified from all four samples tested. Of the 29,489 genes, 28,074 were previously predicted in the lotus reference gene set^27^, while the remaining 1415 genes were not previously included in the lotus reference gene set, thus representing novel genes. Moreover, 24,728, 24,667, 24,782, and 24,641 genes were expressed in LM_S1, LM_S4, WD_S1, and WD_S4 samples, respectively, while 22,800 genes were commonly expressed in all four samples tested. By contrast, 297, 215, 239, and 279 genes were exclusively expressed in LM_S1, LM_S4, WD_S1, and WD_S4 samples, respectively.

### Mining of candidate genes involved in BIA biosynthesis in lotus

**Fig. 3:**
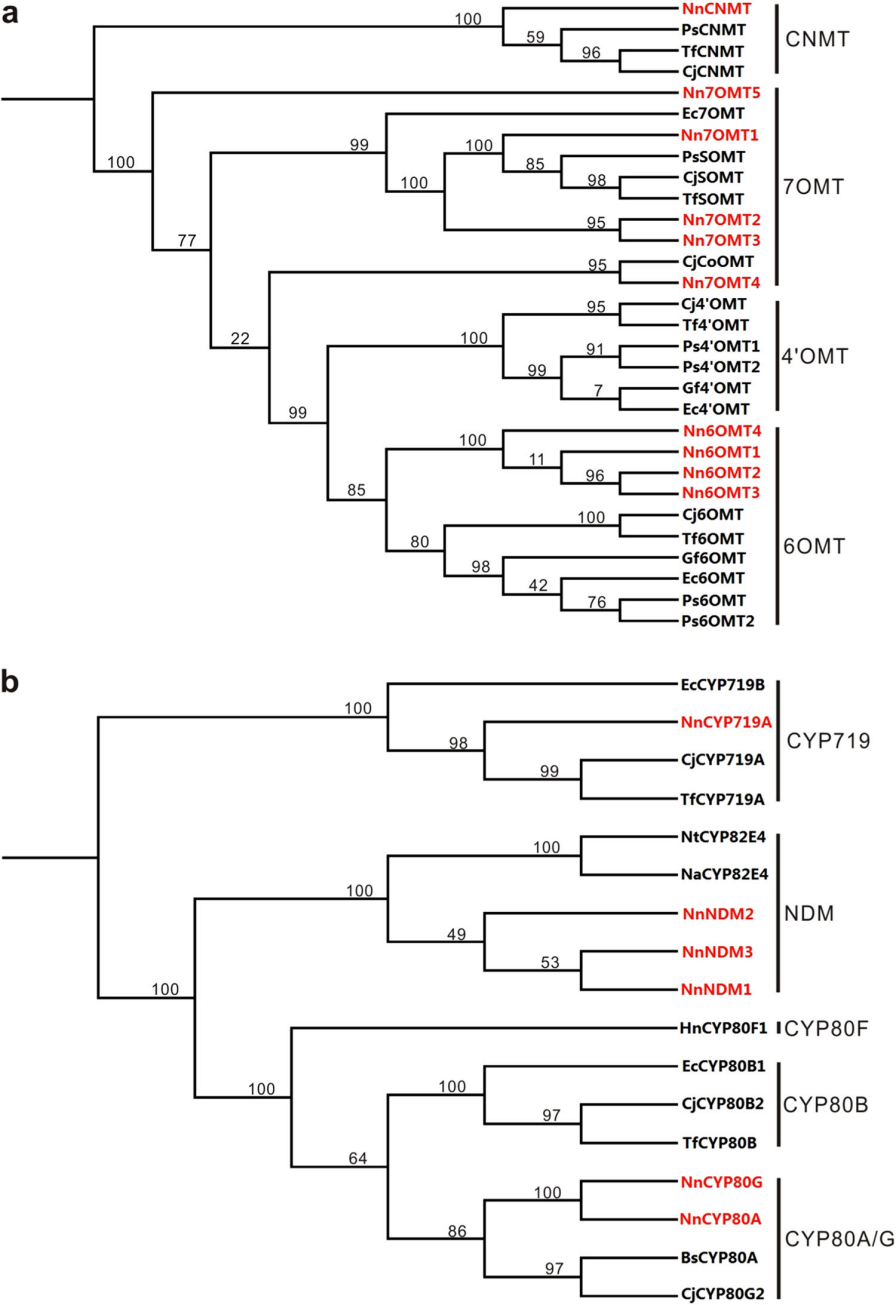
Phylogenetic trees derived from amino acid sequences of genes encoding methyltransferase and CYP protein, respectively, which are expressed in the leaf of lotus. The genes identified in this study are highlighted in red color

To identify BIA biosynthetic pathway genes in lotus, coding DNA sequences of previously reported BIA biosynthesis genes were compared against those of the 29,489 genes expressed in lotus leaf. Initially, we investigated structural genes involved in the biosynthesis of the BIA precursor (*S*)-reticuline. Five, four, and one copies of *TYDC*, *NCS*, and *CNMT* genes, respectively, were identified (Table S2) and the amino acid sequence alignment of each gene family is shown in Supplementary data 1. Seven copies of genes encoding OMT showed high similarity to both *6OMT* and *7OMT* in *Papaver somniferum*^35–37^ and *Coptis japonica*^38^. Phylogenetic analysis showed that all these *OMT* genes were closely related to either the *6OMT* or *7OMT* gene clusters, but distinct from the *4*′*OMT* gene cluster (Fig. 3a). Likewise, three copies of genes encoding BIA-related cytochrome P450 (CYP) were identified, but none of them was phylogenetically close to *CYP80B* genes (Fig. 3b).

Lotus leaf accumulates five types of BIAs, nuciferine, *N*-nornuciferine, *O*-nornuciferine, roemerine, and anonaine, with nuciferine as the predominant type^8^. The conversion of the (*S*)-reticuline precursor to nuciferine consists of at least two reactions, an intramolecular C–C phenol coupling and a 7-*O*-methylation, under the catalyzation of CYP80G^39^ and 7-*O*-methyltransferase^35^, respectively. Likewise, an intramolecular C–C phenol-coupling reaction together with a methylenedioxy bridge-forming reaction catalyzed by CYP719A^40^ are required for the biosynthesis of roemerine. Additional *N*-demethylation and *O*-demethylation reactions that are catalyzed by *N*-demethylase (NDM) or *O*-demethylase (ODM), respectively^41,42^, result in structurally diversified BIAs. Analysis of the expressed genes in the leaf revealed one, three, one, three, and four copies of *CYP80G*, *7OMT*, *CYP719A*, *NDM*, and *ODM* genes, respectively.

The *NnCYP80A* and *NnCYP80G* genes shared approximately 88.8% identity in coding DNA sequence, and they were phylogenetically related to previously reported *CYP80A* and *CYP80G* genes, but separated from the *CYP80B* gene cluster (Fig. 3b). CYP80A is well known to catalyze (*S*)-*N*-methylcoclaurine to form bis-BIAs^43,44^, while CYP80G is involved in the aporphine-type BIA biosynthesis^39^. Lotus embryo and leaf predominantly accumulates bis-BIAs and aporphine-type BIAs, respectively^8^. Hence, *NnCYP80A* and *NnCYP80G* are likely associated with BIA accumulation in the embryo and leaf, respectively. To confirm this hypothesis, we conducted quantitative real-time PCR (qRT-PCR) analysis. The results showed that *NnCYP80A* was highly expressed in the embryo, but with an extremely low expression in the leaf (Fig. S2). In contrast, *NnCYP80G* was highly expressed in the leaf, but its transcripts were nearly undetectable in the embryo. This spatial expression pattern is consistent with the accumulation of different types of BIAs in the embryo and leaf.

### Difference in expression of BIA biosynthetic pathway genes in the leaf between different developmental stages or between cultivars

**Fig. 4:**
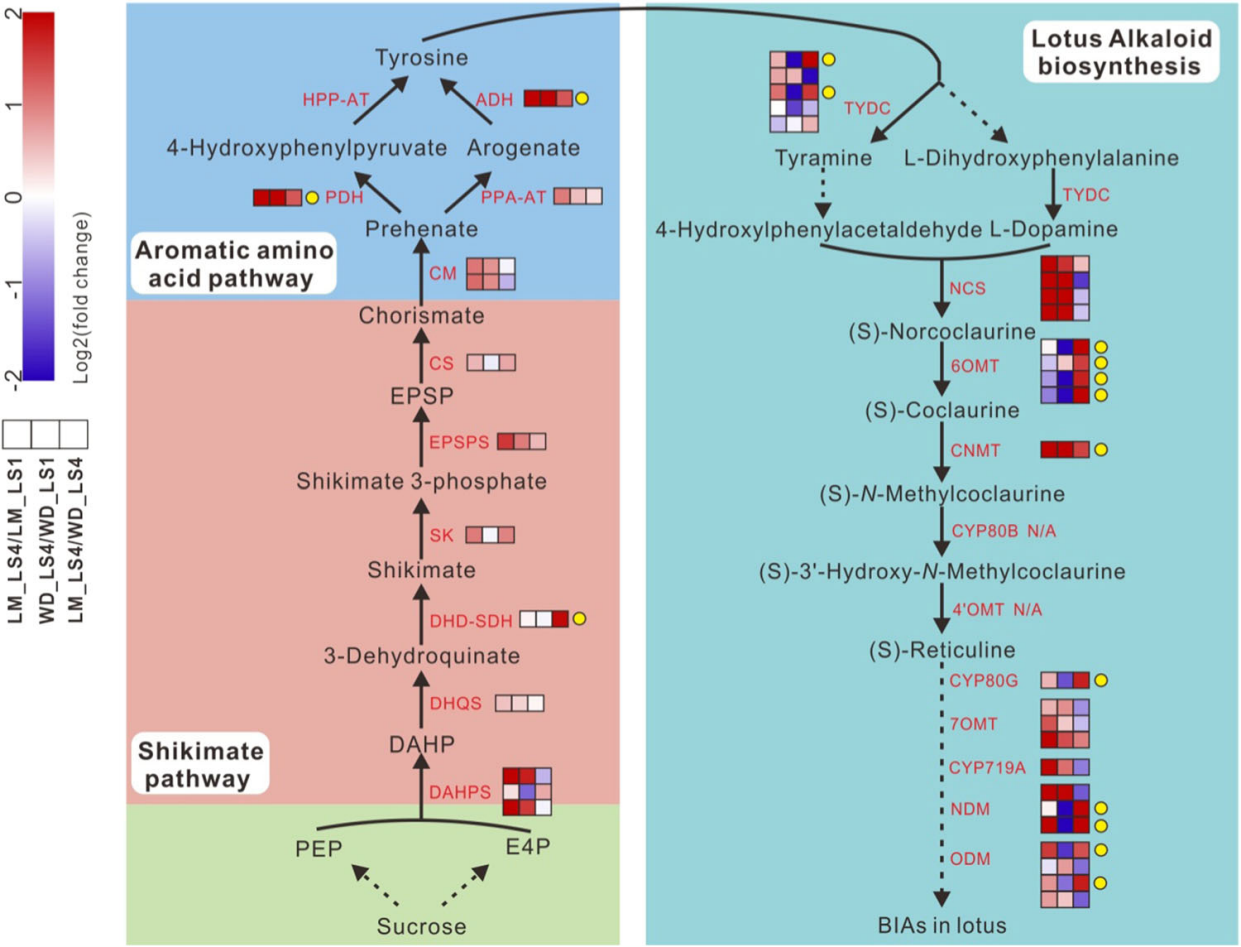
Schematic diagram of fold change in expression level of structural genes in BIA, shikimate, and aromatic amino acid pathways in lotus leaf between two stages of the same cultivar or between cultivars with the same stage 4. Three boxes that are arranged in the same row indicate comparisons of LM_LS4/LM_LS1, WD_LS4/WD_LS1, and LM_LS4/WD_LS4, respectively. The layers of boxes represent different gene copies, which are listed in a numerical order from top to bottom. LM_LS4/LM_LS1 and WD_LS4/WD_LS1 represent comparison of gene expression levels in leaves of cvs. LM and WD, respectively, between stages 1 and 4, while LM_LS4/ WD_LS4 indicates comparison of gene expression levels at stage 4 between cvs. LM and WD. The arrows with broken lines indicate multi-enzymatic reactions. PEP phosphoenolpyruvate, E4P d-erythrose 4-phosphate, DAHPS 3-deoxy-d-*arabino*-heptulosonate 7-phosphate synthase, DHQS 3-dehydroquinate synthase, DHD 3-dehydroquinate dehydratase, SDH shikimate dehydrogenase, SK shikimate kinase, PESPS 5-enolpyruvylshikimate-3-phosphate synthase, CS chorismate synthase, CM chorismate mutase, PPA-AT prephenate aminotransferase, ADH arogenate dehygrogenase, PDH prephenate dehydrogenase, HPP-AT 4-hydroxyphenylpyruvate aminotransferase

To uncover the mechanism underlying leaf alkaloid accumulation, we further investigated the digital expression profiles of BIA biosynthetic pathway genes in leaves of “LM” and “WD” at two developmental stages. For cv. LM, almost all the BIA pathway genes except *NnTYDC5*, *Nn6OMT2*, *Nn6OMT3*, *Nn6OMT4*, *NnCYP80A*, and *NnNDM3* were up-regulated in the leaf at stage 4 compared with stage 1 (Fig. 4). For example, the expression levels of *NnNCS* genes in stage 4 showed 5-fold to 30-fold higher than those in stage 1 (Table S2). The *NnCNMT* gene showed an approximately 9-fold increase in expression level at stage 4 compared with stage 1. In addition, we also analyzed the dynamic expression patterns of genes involved in shikimate and aromatic amino acid pathways, which supply the tyrosine source for BIA biosynthesis. All genes in these two pathways were up-regulated in the leaf of “LM” at stage 4 compared with stage 1. For example, two copies of genes encoding 3-deoxy-d-arabino-heptulosonate 7-phosphate synthase, a rate-controlling enzyme in the shikimate pathway^45^, showed a 4-fold increase at stage 4 compared with stage 1. The gene encoding agrogenate/prephenate that converts arogenate to tyrosine displayed an over 26-fold increase.

For cv. WD, approximately half of the BIA pathway genes were up-regulated or down-regulated in the leaf at stage 4 compared with stage 1. Moreover, eight shikimate related and four aromatic amino acid-related pathway genes were up-regulated and down-regulated, respectively, in the leaf at stage 4 compared with stage 1. Almost all the up-regulated genes in cv. WD had smaller fold-change values than in cv. LM.

In the leaf at stage 4, 16 BIA pathway genes showed higher levels of expression in cv. LM compared with cv. WD, with a 4.9-fold increase on average. However, the remaining 11 genes had lower levels of expression in cv. LM than in cv. WD. Moreover, most genes involved in shikimate and aromatic amino acid pathways were expressed at higher levels in cv. LM than in cv. WD.

In summary, a set of BIA biosynthetic pathway genes were up-regulated in the leaf at stage 4 compared with stage 1 in both cultivars tested. In the leaf at stage 4, multiple BIA biosynthetic pathway genes also showed higher level expression in high-BIA cultivar than in low-BIA cultivar. These findings suggest the possibility of transcriptional regulation mechanism controlling BIA biosynthesis in lotus.

### Mining of candidate regulatory genes controlling BIA accumulation in lotus

As mentioned above, the high-BIA “LM” and the low-BIA “WD” cultivars showed a significant difference in alkaloid content of leaves at stage 4, but with a relatively similar low content at stage 1. Moreover, “LM” showed a great variation of alkaloid content in leaves between stages 1 and 4. Thus, comparative analysis of two paired transcriptome profiles, LM_S4 vs. LM_S1 and LM_S4 vs. WD_S4, was conducted to identify differentially expressed regulatory genes related to BIA accumulation. However, the comparison of WD_S4 vs. WD_S1 was not included in this study because “WD” had relatively low levels of BIA accumulation in the leaf, which is probably due to mutation of key regulatory gene(s) involved in BIA biosynthesis.

**Fig. 5:**
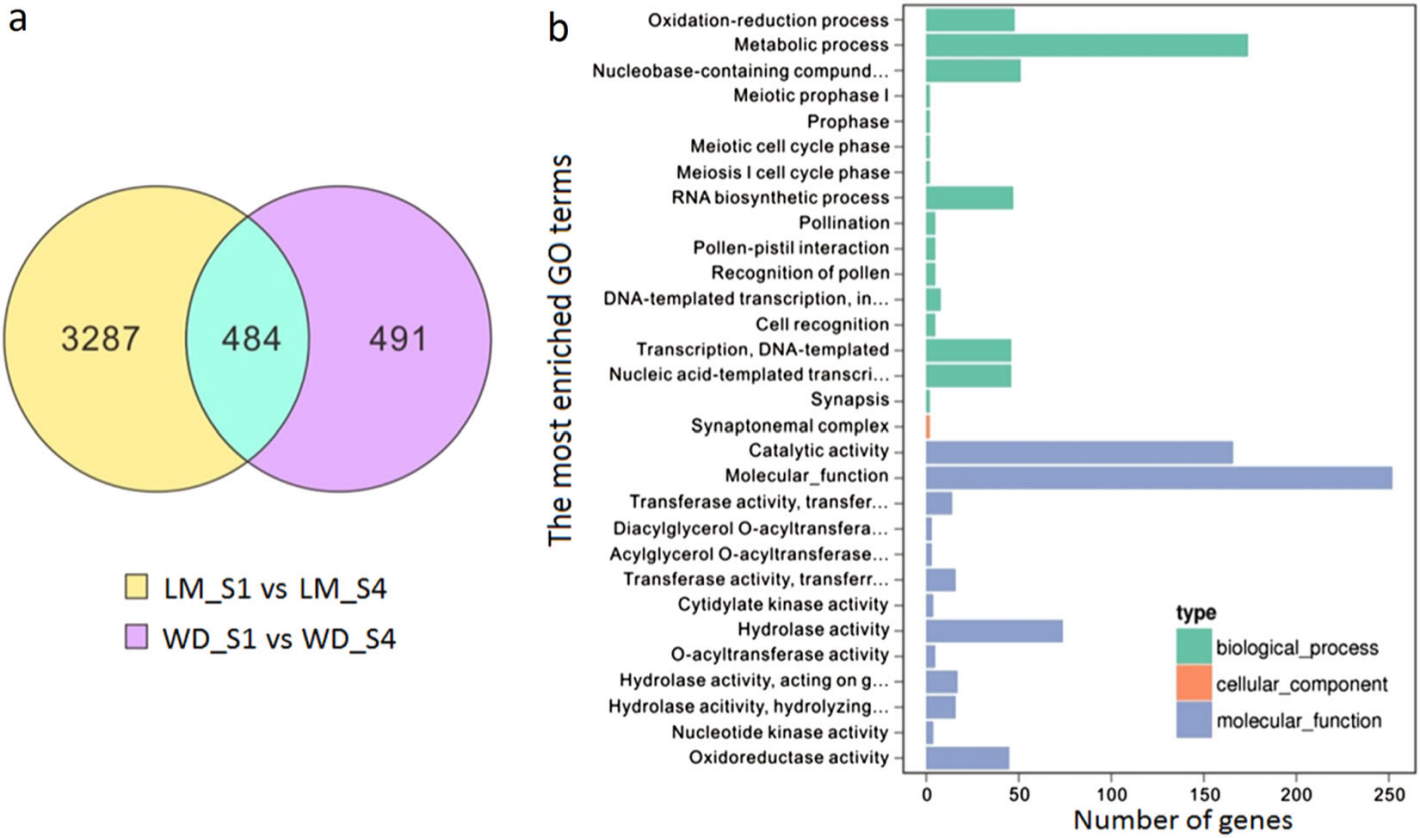
Identification of candidate transcription factors regulating BIA accumulation in lotus. **a** Venn diagrams showing the numbers of genes commonly and exclusively up-regulated or down-regulated in leaves of each cultivar tested between stages 1 and 4. **b** Gene ontology (GO) enrichment analysis of the 484 genes commonly differentially expressed in leaves of cultivars LM and WD between stages 1 and 4

A total of 4072 genes were identified to be differentially expressed in leaves of cv. LM, with 2145 and 1927 up-regulated and down-regulated, respectively, when stage 4 was compared with stage 1. Similarly, 976 genes were differentially expressed in leaves at stage 4 between cvs. LM and WD, with 523 and 453 genes up-regulated and down-regulated, respectively. Of these differentially expressed genes (DEGs), 484 (Supplementary data 2) were commonly up-regulated or down-regulated among the two comparisons (Fig. 5a), and they were deemed to contain candidate regulatory genes for BIA biosynthesis in the leaf. GO enrichment analysis indicated that all the 484 DEGs had one or more GO annotations, including 450 biological process, 2 cellular component, and 619 molecular function terms (Fig. 5b).

Of the 484 differentially expressed genes, 24 were TFs encoding AP2/ethylene-responsive factor (ERF), WRKY, MYB, helix–loop–helix (bHLH), NAC, and zinc-finger protein (Table 2). Previous studies showed that several TFs, such as bHLH, WRKY, and ORCA3, are involved in the regulation of BIA biosynthesis in plants^46,47^. Therefore, sixteen TFs, including six *NnWRKYs*, five *NnERFs*, four *NnMYBs*, and one *NnbHLH*, were deemed to be candidate regulators responsible for BIA biosynthesis in lotus.

**Table 2:** Transcription factors potentially involved in the regulation of BIA biosynthesis in lotus

Number	Name	ID	Type	Fold change*		
				LM_S4/LM/S1	WD_S4/WD_S1	LM_S4/WD_S4
1	*NnERF81*	104599450	AP2/ERF	1.87	0.41	3.21
2	*NnRAV1*	104603036	AP2/ERF	12.23	1.27	3.65
3	*NnRAP23*	104606214	AP2/ERF	3.82	0.89	2.97
4	*NnERF92*	104610030	AP2/ERF	3.15	0.69	2.78
5	*NnERF2*	104610490	AP2/ERF	3.07	0.69	2.41
6	*NnWRKY6*	104588301	WRKY	4.15	0.66	2.21
7	*NnWRKY701*	104593755	WRKY	3.67	0.49	3.96
8	*NnWRKY702*	104594468	WRKY	1.91	0.73	2.47
9	*NnWRKY31*	104594045	WRKY	75.32	3.77	2.85
9	*NnWRKY401*	104603312	WRKY	4.73	0.35	3.00
10	*NnWRKY402*	104610723	WRKY	13.44	1.12	2.24
11	*NnMYB6*	104589500	MYB	5.92	1.25	2.39
12	*NnMYB113*	104599741	MYB	3.94	3.23	2.18
13	*NnMYB12*	104605119	MYB	4.73	1.22	4.03
14	*NnMYB4*	104610016	MYB	14.62	0.55	2.94
15	*NnZHD11*	104587730	Zinc-finger	2.54	2.98	2.04
16	*NnZAT8*	104594613	Zinc-finger	2.56	0.19	3.35
17	*NnZAT10*	104608146	Zinc-finger	2.34	0.47	2.02
18	*NnbHLH1*	104606713	bHLH	3.27	3.38	13.41
19	*NnNAC72*	104612551	Nac	26.85	2.11	3.97
20	*NnPHRF1*	104588985	PHD	1.92	0.76	2.36
22	*NnDIV*	104589256	DIVARICATA	2.30	0.34	2.42
23	*NnAPRR5*	104590049	PRR	3.16	0.90	3.28
24	*NnJM706*	104588821	JMJ	1.75	0.761	2.82

^*^ LM_LS4/LM_LS1 and WD_LS4/WD_LS1 represent fold-change values derived from comparison of gene expression levels in leaves of cvs. LM and WD, respectively, between stages 1 and 4, while LM_LS4/WD_LS4 indicates fold-change value for the comparison of gene expression levels at stage 4 between cvs. LM and WD.

### Expression consistency between BIA biosynthetic pathway genes and their candidate regulators in the leaf of two tested cultivars

**Fig. 6:**
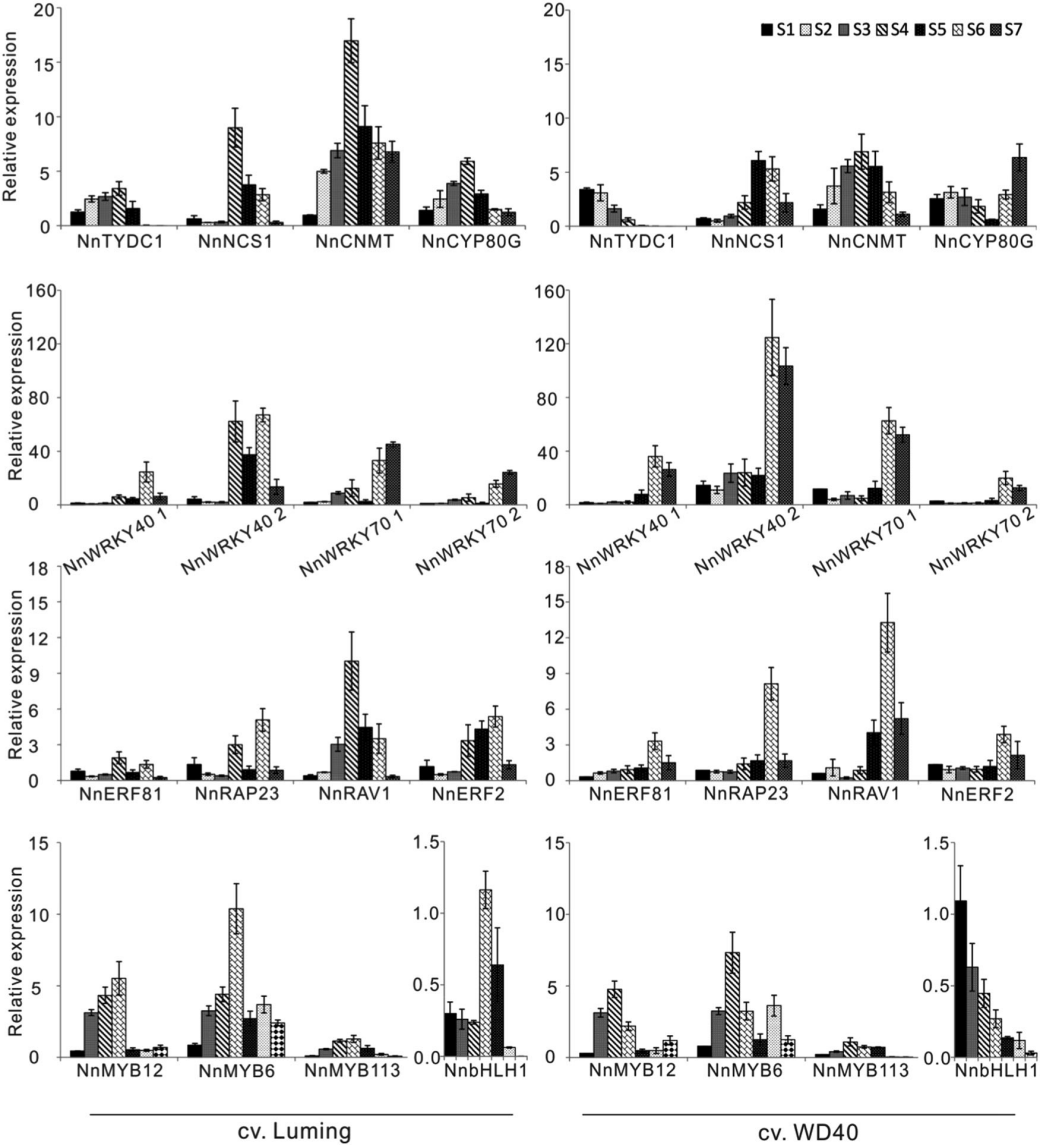
qRT-PCR analysis of BIA biosynthetic pathway genes and candidate regulators in leaves of two lotus cultivars throughout the whole developmental stages

To verify regulatory role of the above-mentioned candidate TFs, their expression profiles in the leaf throughout whole developmental stages were compared with those of BIA biosynthetic pathway genes. Overall, all the four structural genes tested, *NnTYDC1*, *NnNCS1*, *NnCNMT*, and *NnCYP80G*, showed higher levels of expression in the leaf of cv. LM compared with cv. WD (Fig. 6a). The expression levels of all the tested structural genes in the leaf of cv. LM increased stably during the early stages, reached a peak at stage 4, and decreased gradually during the later stages. Similar expression profile was also observed for structural genes *NnNCS1* and *NnCNMT* in cv. WD. However, the expression level of *NnTYDC1* showed a decrease in the leaf of cv. WD throughout the whole developmental stages, with a highest expression at stage 1. By contrast, the expression level of *NnCYP80G* showed a peak at stage 2, and decreased until stage 5, followed by a substantial increase during the last two stages. For easy description, the expression profile of the structural genes in cv. LM was designated the classic upside-down V pattern.

Of the 16 candidate regulators, four, *NnERF92*, *NnWRKY6*, *NnWRKY31*, and *NnMYB4*, showed a very low expression in leaves of two cultivars throughout the whole development stages, suggesting that they play little role in the regulation of BIA accumulation. Three MYB genes, *NnMYB12*, *NnMYB6*, and *NnMYB113*, had the classic upside-down V pattern of expression in both “WD” and “LM,” with a peak at stage 4 (Fig. 6b). This was consistent with the expression profile of all the four structural genes tested in cv. LM and two structural genes *NnNCS1* and *NnCNMT* in cv. WD. Two genes, *NnbHLH1* and *NnRAV1*, showed the classic upside-down V pattern of expression in cv. LM, but not in cv. WD. However, the rest candidate regulators showed no classic upside-down V pattern of expression in either “LM” or “WD.”

The above results suggest that five TFs, *NnMYB12*, *NnMYB6*, *NnMYB113, NnbHLH1*, and *NnRAV1*, are deemed to be candidate regulators of BIA accumulation in lotus.

### Functional analysis of candidate TFs involved in BIA regulation using dual luciferase assay

**Fig. 7:**
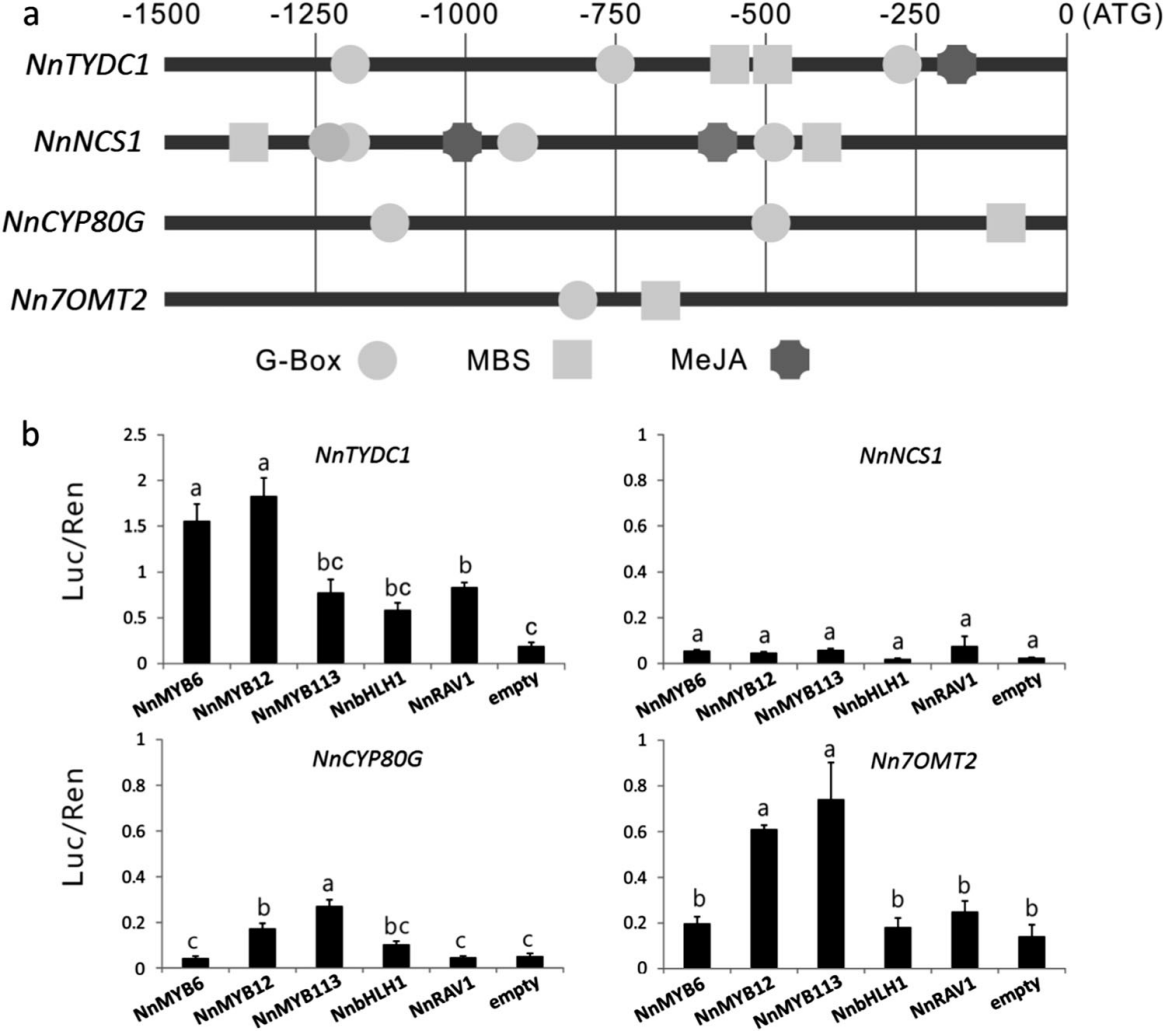
Relationship between regulatory genes and BIA biosynthetic pathway genes in lotus. **a** *Cis*-elements in the promoter regions 1.5-kb upstream of the translation initiation codon of BIA biosynthetic pathway genes in lotus. ATG, start codon. MeJA methyl jasmonate, MBS MYB binding site. **b** Estimation of effect of TFs on activation of the promoter of BIA pathway genes using transient dual luciferase assay. Error bars show SE of three biological replicates. Different lowercase letters indicate significant difference at *P* < 0.05

To gain an insight into regulatory mechanism controlling BIA biosynthesis in lotus, *cis*-regulatory elements were scanned in the promoter region of four BIA structural genes, including *NnTYDC1*, *NnNCS1*, *NnCYP80G*, and *Nn7OMT2*. A 1.5-kb DNA fragment upstream of translational start codon was scanned using the PlantCARE software^48^. All the promoters tested contained putative *cis*-elements, MBS and G-box, which are the binding sites of MYB and AP2/ERF TFs, respectively (Fig. 7a). Two genes, *NnTYDC1* and *NnNCS1*, contained one or two, respectively, putative MeJA-response *cis*-elements (MeJA), the possible binding site of bHLH and AP2/ERF TFs. These results suggest that the five regulators mentioned above, *NnMYB12*, *NnMYB6*, *NnMYB113*, *NnbHLH1*, and *NnRAV1*, could bind to the promoters of BIA pathway genes to induce their transcription, resulting in the accumulation of BIAs in lotus leaf.

To validate whether these regulators had direct activation on transcription of structural genes, dual luciferase assay was further carried out (Fig. 7b). Of the three MYB regulators, *NnMYB12* was well able to activate the promoters of three structural genes, *NnTYDC1*, *NnCYP80G*, and *Nn7OMT2*. *NnMYB6* was well able to activate the promoter of *NnTYDC1*, while *NnMYB113* showed a relative strong activation on the promoters of *NnCYP80G* and *Nn7OMT2*. *NnRAV1* was able to activate the *NnTYDC1* promoter, but *NnbHLH1* showed no activation on the promoters of all structural genes tested. These results indicate that the three MYB TFs are likely to play an important role in BIA regulation.

In addition, NCS is crucial for BIA accumulation as it catalyzes condensation of dopamine and 4-HPAA to form (*s*)-norcoclaurine, a central precursor of BIAs. However, dual luciferase assay showed that the *NnNCS1* promoter could not be activated by all the five candidate regulators. Interestingly, *NnbHLH1* co-infiltrated with *NnRAV1* showed a strong activation on the *NnNCS1* promoter (Fig. S3), suggesting a potential interaction between the *NnbHLH1* and *NnRAV1* regulators. In contrast, *NnbHLH1* co-infiltrated with *NnMYB113* showed no activation on the *NnNCS1* promoter although the interaction between MYB and bHLH TFs has been well characterized. In addition, *NnbHLH1* co-infiltrated with *NnRAV1* or *NnMYB113* did not increase significantly the activation on the *NnTYDC* promoter compared with those infiltrated with *NnbHLH1*, *NnRAV1*, or *NnMYB113* alone.

Taken together, the results presented above indicate that all the five candidate regulators may act in an independent or cooperative manner to induce the transcription of BIA biosynthetic pathway genes, resulting in a complex network of transcriptional regulation controlling BIA accumulation in lotus.

## Discussion

### The BIA biosynthetic pathway and its regulation in lotus

(*S*)-reticuline is well known to be the common precursor to BIA alkaloids, such as morphine, sanguinarine, papaverine, and berberine. However, an exception has been noted in dimeric bis-BIA alkaloids such as berbamunine in *Berberis stolonifera*, which result from the regioselective and stereoselective oxidative carbon–oxygen phenol coupling of (*S*)-*N*-methylcoclaurine under the catalyzation of CYP80A^43^. In this study, a *CYP80A* homolog *NnCYP80A* was also identified in lotus leaf transcriptome, although its expression was extremely low. However, the *NnCYP80A* gene is highly expressed in lotus embryo. In lotus, bis-BIA alkaloids are predominantly accumulated in embryos, but almost undetectable in the leaf. These results suggest that (*S*)-*N*-methylcoclaurine is the precursor to bis-BIAs in lotus embryos. However, more studies are still needed to address whether this reaction is catalyzed by *NnCYP80A*.

**Fig. 8:**
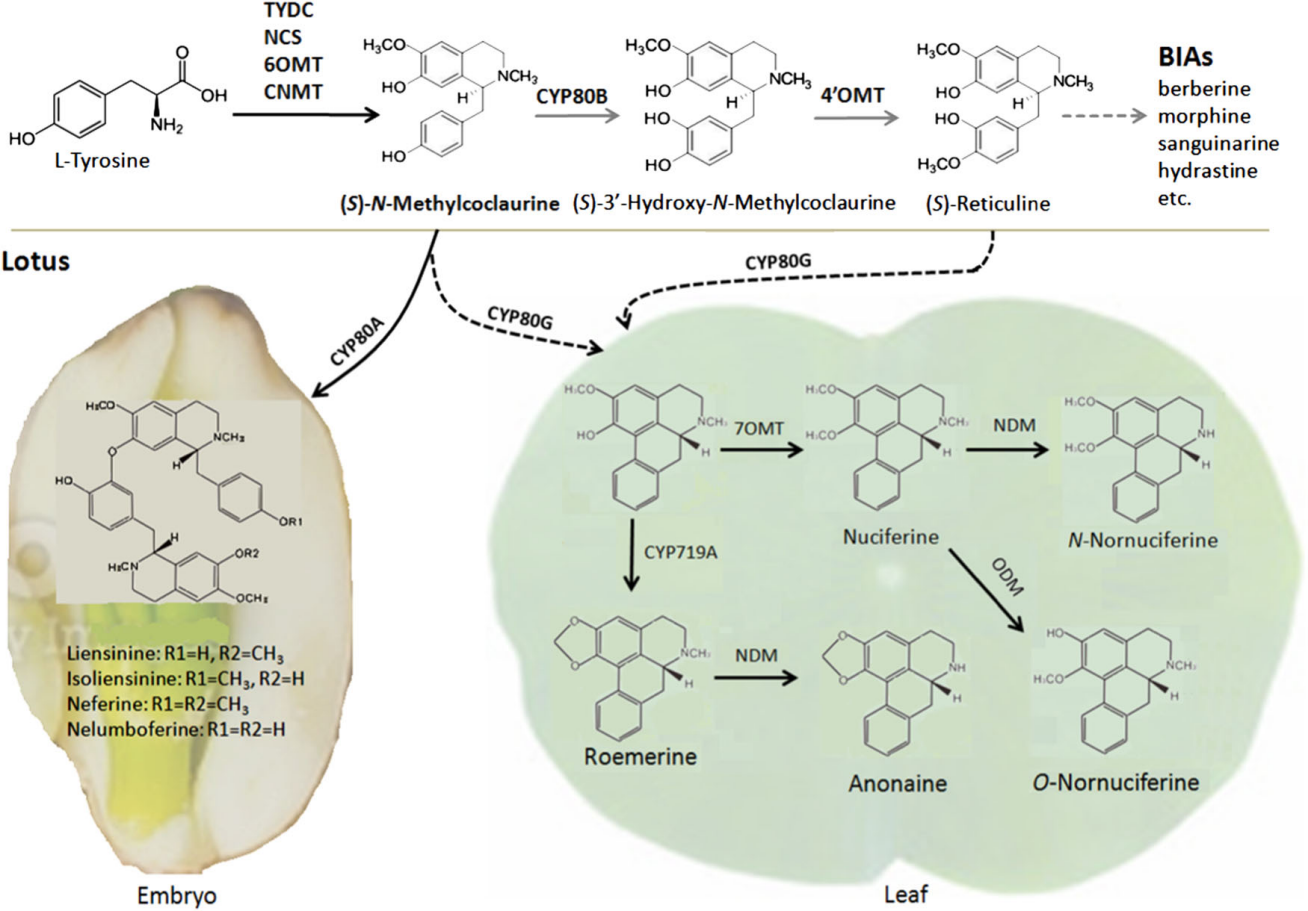
Schematic diagram of a proposed biosynthetic pathway of BIAs derived from the precursor (*S*)-*N*-methylcoclaurine in lotus. Lotus leaf accumulates aporphine-type BIAs, while embryo accumulating bis-BIAs. The gray line indicates the common biosynthetic pathway of BIAs derived from the precursor (*S*)-reticuline. The black dash line indicates the potential biosynthetic pathway of BIAs in the leaf. Dotted lines represent multiple enzymatic steps

Conversion of (*S*)-*N*-methylcoclaurine to (*S*)-reticuline requires two enzymatic steps catalyzed by CYP80B and 4′OMT^49^. Although OMTs involved in BIA biosynthesis have extensive amino acid homology, they can be divided into distinct clades, such as 6OMT, 4′OMT, 7OMT, and 9OMT, based on phylogenetic analysis^50,51^. Here, nine *OMT* genes were identified in the lotus leaf transcriptome, but none of them was phylogenetically related to previously reported 4′*OMT* genes. Similarly, two *CYP80* genes were found in the lotus leaf transcriptome, but neither of them appeared in the *CYP80B* cluster. In addition, CYP80B and 4′OMT are responsible for hydroxylation and methylation at the C-3′ and C-4′ position, respectively^52,53^. However, the aporphine-type BIAs in lotus leaf do not contain any hydroxyl or methyl groups at the C-4′ and C-3′ position, which suggests that the two enzymatic steps catalyzed by CYP80B and 4′OMT are not crucial for the biosynthesis of aporphine-type BIAs in lotus. Given the finding that neither *CYP80B* nor *4*′*OMT* genes are found in the leaf transcriptome, we speculate that (*S*)-*N*-methylcoclaurine may serve as the precursor to both aporphine and bio-BIA alkaloids in lotus. However, one thing to keep in mind is that the lotus leaf transcriptome may contain other genes with functionality similar to *CYP80B* and *4*′*OMT*, resulting in a possible pathway that uses (*S*)-reticuline as precursor for BIA synthesis, similar like most of other BIA-producing plants such as opium poppy and California poppy. Therefore, we propose a model to illuminate the BIA biosynthetic pathway in lotus (Fig. 8). In addition, it is worth noting that conversion of (*S*)-*N*-methylcoclaurine or (*S*)-reticuline to lotus aporphine alkaloids requires enzymatic steps, dehydroxylation at the C-4′ position, and/or demethylation at C-3′ position. Thus, more studies are still needed to clarify enzymatic reactions that convert (*S*)-*N*-methylcoclaurine or (*S*)-reticuline into aporphine alkaloids in lotus.

Increasing evidences show that alkaloid accumulation is controlled by TFs such as *WRKY*, *bHLH*, and *ERF* at the transcriptional level^54–58^. Such transcriptional regulation was also observed in our study, with a set of BIA biosynthetic pathway genes showing higher expression in the leaf of high-BIA cv. LM compared with low-BIA cv. WD. Two TFs, *NnbHLH1* and *NnRAV1*, both show an expression profile that is consistent with those of BIA structural genes in the leaf of cv. LM, but not in the leaf of cv. WD. The G-box or MeJA binding motifs for *NnbHLH1* and *NnRAV1* were found in the promoter of BIA structural genes. Dual luciferase assay shows that *NnbHLH1* and *NnRAV1* may be interacted with each other to form a transcriptional regulation for alkaloid biosynthesis in lotus.

The *WRKY1* gene has been reported to control BIA accumulation by activating the *4*′*OMT* gene in *C. japonica*^59^ and California poppy^60^. In this study, two homologs of *CrWRKY1*, *NnWRKY701*, and *NnWRKY702*, together with four additional WRKY TFs, were identified in lotus leaf transcriptome. However, none of these *NnWRKY* TFs shows a consistent expression profile with BIA structural genes in the leaf of both “LM” and “WD.” It is unclear whether this inconsistency is attributed to the lack of the *4*′*OMT* gene in the leaf transcriptome. Interestingly, three MYB regulators, *NnMYB6*, *NnMYB12*, and *NnMYB113*, show a consistent expression profile in the leaf of both “LM” and “WD” with BIA structural genes. The activation of these *MYB* genes on BIA structural genes was confirmed by dual luciferase assay. In addition, MYB binding motifs have been found in the promoters of all the four BIA structural genes tested. Thus, these results suggest that *NnMYB6/NnMYB12/NnMYB113*, like *NnbHLH1* and *NnRAV1*, have a potential to induce BIA structural gene transcription by binding the MBS motif in their promoters. To our knowledge, this study reveals for the first time that MYB TFs are involved in the regulation of BIA accumulation. In addition, recent studies have shown that MYB TFs can interact with AP2/ERF TFs to regulate anthocyanin and lignin biosynthesis in fruits^61,62^. Thus, it is worthy of further study to ascertain whether *NnMYB6/NnMYB12/NnMYB113* regulate lotus BIA biosynthesis through interacting with AP2/ERF TFs in lotus.

*NnbHLH1* alone is unable to induce BIA structural gene transcription, but it may interact with *NnRAV1* to induce the transcription of *NnNCS1*, which cannot be activated by *NnMYB* genes. Moreover, *NnRAV1* alone shows a relative weak activation on the *NnTYDC* transcription. Unlike *NnbHLH1* and *NnRAV1*, the *NnMYB* genes are able to individually activate three out of the four BIA structural genes tested. These results not only show that *NnMYBs* are likely to key regulators controlling BIA accumulation in lotus but also suggest a complex network of transcriptional regulation involved in lotus BIA biosynthesis. Moreover, a bHLH TF *CrMYBC2* is well known to function as the major activator of the expression of the AP2/ERF-domain TF *ORCA3* in *Catharanthus roseus*^63^. In this study, the G-box of bHLH binding motif was found in the promoter of *NnMYB6/NnMYB12* and *NnRAV1*. It is worthy of further study to clarify whether *NnbHLH1* has ability to active transcription of *NnMYB6/NnMYB12* and *NnRAV1* by binding the G-box motif in their promoters, resulting in hierarchical interaction between regulators involved in BIA accumulation.

### Tandem duplication of BIA biosynthesis genes and its impact on structural diversity of BIAs in lotus

Gene duplication is a major driving force for recruitment of genes involved in secondary metabolism, which contributes to structural diversify of secondary metabolites in plants. Duplicated genes involved in secondary metabolism often appear to cluster within the plant genome^64^, and tandem duplication is frequently observed for genes involved in biosynthesis of secondary metabolites, such as flavonoids^65^, volatiles^66^, caffeine^67^, and BIA^68^.

Our recent study reveals a cluster of five *NnNCS* genes within an 83-kb region on linkage group 8 of sacred lotus^30^. Only one member, *NnNCS1* out of the five *NnNCS* genes, was expressed in the leaf, which suggests that they have undergone divergence in expression after duplication^69^. Here, three more clusters of BIA structural genes were also observed. First, two CYP80 genes, *NnCYP80A* and *NnCYP80G*, are located closely to each other within a 20-kb region on scaffold 12^27^, which suggests that they are evolved from a common ancestor. As mentioned above, their spatiotemporal expression results in difference in alkaloid component between organs in lotus. This indicates that *NnCYP80A* and *NnCYP80G* have functionally diverged after duplication. Second, two *6OMT* genes, *Nn6OMT2* and *N6OMT3*, are located in a 42-kb region on scaffold 432^27^. These two *OMT* genes are both expressed in the leaf with similar expression level. Last, three ODM genes, *NnODM2*, *NnODM3*, and *NnODM4*, are tightly clustered within an 85-kb region on scaffold 80^27^. These three *ODM* genes are all expressed in the leaf, but show a divergence in expression level, with *NnODM2* having the highest expression level, followed by *NnODM3* and *NnODM4*. Thus, it seems that the clustered *Nn6OMT* and *NnODM* genes may be functionally redundant.

In summary, tandem duplication occurs often among BIA structural genes, and the duplicated genes may have undergone divergence in enzymatic activity and/or expression in lotus. The functional divergence of two duplicated *CYP80* genes, *NnCYP80A* and *NnCYP80G*, play an important role in structural divergence of two groups of alkaloids in lotus, aporphine-type BIAs and bis-BIAs. In addition, lotus leaf is becoming more and more attractive because it provides a resource of the bioactive BIA ingredients with diversified health benefits. Our study reveals the biosynthetic pathway and transcriptional regulation of BIAs in lotus, which not only provides a deeper understanding of BIA biosynthesis in plants but will also be helpful for development of new cultivars with high level of BIAs in lotus breeding programs.

## Electronic supplementary material

Supplementary file modified

Suppementary data 1

Supplementary data 2
