# Supplementary material for: Investigation of benzylisoquinoline alkaloid biosynthetic pathway and its transcriptional regulation in lotus
Source: Hortic Res. 2018 Jun 1;5:29. doi: 10.1038/s41438-018-0035-0 (PMC5981371; doi:10.1038/s41438-018-0035-0)
Supplement: Supplementary file 1 — Supplementary file modified [file 41438_2018_35_MOESM1_ESM.doc]

Table S1. Primers used qRT-PCR and dual luciferase assay

| No | Type | Name | Sequence (5’-3’) |
| --- | --- | --- | --- |
| 1 | qRT-PCR | NnTYDC1_F | GCAAGACGTGCAGAAAGAAATTATCC |
| 2 |  | NnTYDC1_R | GCAGCAGGCGAAGACATCCAA |
| 3 |  | NnNCS1_F | GAGCTGCCCAGACTCTTCGTC |
| 4 |  | NnNCS1_R | TACCTTCTCCCGGTGTTGATG |
| 5 |  | NnCNMT_F | CCATCAAGACCGACTTACCAA |
| 6 |  | NnCNMT_R | TCACAGTACAGCTCCAGCATAG |
| 7 |  | NnCYP80G_F | AGCGTCCGAATTAAGGGCTAT |
| 8 |  | NnCYP80G_R | ATCAGTTCCGATACTTTCTCCTCT |
| 9 |  | NnACTIN_F | CTCCGTGTTGCCCCTGAAG |
| 10 |  | NnACTIN_R | CCAGCAAGGTCCAACCGAAG |
| 11 |  | NnWRKY701_F | CAGGAATGCAAAGAGGTGGAGA |
| 12 |  | NnWRKY701_R | ACCTGAGTACACGCCCGAGAT |
| 13 |  | NnWRKY702_F | CAGGAATGCAAAGAGGTGGAGA |
| 14 |  | NnWRKY702_R | ACCTGAGTACACGCCCGAGAT |
| 15 |  | NnWRK31_F | GATGTCAATGGCGAAAGTATGG |
| 16 |  | NnWRK31_R | GGAGGGAGTGGATGGTTATGGT |
| 17 |  | NnWRK40_1_F | GGGGAGGAATCACTCGGTAAA |
| 18 |  | NnWRK40_1_R | TGAAGCCCGTTTCGTTGTTCT |
| 19 |  | NnWRK40_2_F | TTGGTAGCGACATACGAAGGC |
| 20 |  | NnWRK40_2_R | TTGGGTGAGATCAAGTGTTATGGTT |
| 21 |  | NnWRKY6_F | GACGACGGGATTTCACCTAATG |
| 22 |  | NnWRKY6_R | CTTTCAGCCTTGCGGTTCTGTT |
| 23 |  | NnERF81_F | GGGGAAGATACGCTGCCGAGAT |
| 24 |  | NnERF81_R | AAGGACAAACCAGTTGACCGTGG |
| 25 |  | NnRAV1_F | CGCCGTCACCAACTTCAAGCC |
| 26 |  | NnRAV1_R | ACCAAAGCCGCCACCACCTCT |
| 27 |  | NnRAP23_F | TGACAGGGAAGCAAGGAAGATAAG |
| 28 |  | NnRAP23_R | TCAGATGAGCGGGAAATCCAAA |
| 29 |  | NnERF92_F | CTGCCAGAGTAAAGGATGAAGAAG |
| 30 |  | NnERF92_R | CAGCCTCCGCACTGTCAAACG |
| 31 |  | NnERF2_F | TGTTCAGGCGAGGAATCAAGG |
| 32 |  | NnERF2_R | ACATTACAAGCGTCACGGAGCAT |
| 33 |  | NnMYB06_F | CAGCAGCAACAGCAATACTAACAA |
| 34 |  | NnMYB06_R | CAGGATGACTTCTCCCACGAT |
| 35 |  | NnMYB113_F | TAGGAAAGGTTGCTGGACTGAA |
| 36 |  | NnMYB113_R | CGCTTAATGTTGGGACGAAGATAGT |
| 37 |  | NnMYB12_F | ATGGACTTGGTCAAGATGGGTG |
| 38 |  | NnMYB12_R | TCTCAAGGGTCGTCGGTGTAA |
| 39 |  | NnbHLH1_F | CGACCGTGTCAACGTGTCTTTA |
| 40 |  | NnbHLH1_R | ACCCATCGGGAATCTTGCTTT |
| 41 | dual luciferase assay | pNnTYDC1_F | GGCAGTTTAGGTTTCCATCAG |
| 42 |  | pNnTYDC1_R | GCTGGAGATGGAGATGGAGATG |
| 43 |  | pNnNCS1_F | ACACGCTATCACAAACCTACCT |
| 44 |  | pNnNCS1_R | CTTTTTCTTCGTTGGAAGGATGCA |
| 45 |  | pNnCYP80G_F | ATATAAGGGTCAATTTATGTGGA |
| 46 |  | pNnCYP80G_R | TAGAGTCTATAGAGAAGATGTAGTAC |
| 47 |  | pN7OMT_F | TCGGACTGGAAACTGAGAACC |
| 48 |  | pNn7OMT_R | GGTTGTTCTCTTTTTTAGGACTGC |
| 49 |  | NnMYB6_IF | CGGAATTCATGGGGAGACCTCCTTGCTG |
| 50 |  | NnMYB6_IR | GCTCTAGATCAGAATATTGGAGACAATTCCAC |
| 51 |  | NnMYB12_IF | CGGAATTCATGGGAAGGGCGCCGTGTTG |
| 52 |  | NnMYB12_IR | GCTCTAGATCAAGAAAGGAGCCAAGCAGA |
| 53 |  | NnMYB113_IF | CGGAATTCATGGGTAGGATGAAAAATTATAGGA |
| 54 |  | NnMYB113_IR | GCTCTAGATTATTCACTTAACAAACTCCATATTTC |
| 55 |  | NnbHLH1_IF | CGGAATTCATGACAATTGAATGGAGCGATTG |
| 56 |  | NnbHLH1_IR | GCTCTAGATCAGTCGAGCTGATCAAAGGA |
| 57 |  | NnRAV1_IF | CGGAATTCATGGAGGGAAGTTGCATCGATGA |
| 58 |  | NnRAV1_IR | GCTCTAGATTACAAAGCTCCTACGACGCGTT |

Table S2 Transcripts and fold changes of genes involved in the benzylisoquinoline alkaloid pathway, the shikimate pathway, and the aromatic amino acid pathway

| Number | Name | ID / Scaffold in the genome | Fold change | | |
| --- | --- | --- | --- | --- | --- |
|  |  |  | LM_S4/LM_S1 | WD_S4/WD_S1 | LM_S4/WD_S4 |
| 1 | *NnTYDC1* | 104588789 / 217 | 1.51 | 0.08 | 13.89 |
| 2 | *NnTYDC2* | 104593300 / 7 | 1.58 | 1.48 | 0.08 |
| 3 | *NnTYDC3* | 104610815 / 101 | 2.15 | 0.14 | 3.30 |
| 4 | *NnTYDC4* | 104612241 / 121 | 1.00 | 0.34 | 0.69 |
| 5 | *NnTYDC5* | 104600990 / 29 | 0.74 | 0.97 | 1.48 |
| 6 | *NnNCS1* | 104590414 / 325 | 9.49 | 3.37 | 1.42 |
| 7 | *NnNCS3* | 104609606 / 86 | 11.78 | 11.66 | 0.34 |
| 8 | *NnNCS4* | 104590417 / 325 | 34.18 | 8.22 | 0.69 |
| 9 | *NnNCS7* | 104590419 / 325 | 5.57 | 10.02 | 0.73 |
| 10 | *Nn6OMT1* | 104587969 / 193 | 1.09 | 0.12 | 7.70 |
| 11 | *Nn6OMT2* | 104590844 / 432 | 0.80 | 1.22 | 2.97 |
| 12 | *Nn6OMT3* | 104590845 / 432 | 0.56 | 0.11 | 3.31 |
| 13 | *Nn6OMT4* | 104607696 / 69 | 0.47 | 0.06 | 5.01 |
| 14 | *NnCNMT* | 104600628 / 28 | 9.26 | 4.12 | 2.75 |
| 15 | *Nn7OMT1* | 104586605 / 162 | 1.40 | 1.82 | 0.52 |
| 16 | *Nn7OMT2* | 104585771 / 144 | 2.64 | 1.22 | 0.75 |
| 17 | *Nn7OMT3* | 104601399 / 31 | 7.37 | 2.61 | 1.97 |
| 18 | *NnCYP719A* | 104604446 / 2 | 3.86 | 2.31 | 0.49 |
| 19 | *NnCYP80G* | 104595105 / 12 | 1.56 | 0.39 | 3.40 |
| 20 | *NnCYP80A* | 104595104 / 12 | 0.44 | 0.03 | 4.88 |
| 21 | *NnODM1* | 104594902 / 11 | 152.43 | 23.09 | 0.39 |
| 22 | *NnODM2* | 104608961 / 80 | 1.08 | 0.24 | 13.75 |
| 23 | *NnODM3* | 104608960 / 80 | 1.86 | 0.81 | 3.35 |
| 24 | *NnODM4* | 104608959 / 80 | 1.62 | 1.33 | 0.75 |
| 25 | *NnNDM1* | 104505945 / 0 | 4.87 | 0.24 | 4.73 |
| 26 | *NnNDM2* | 104591918 / 5 | 3.23 | 0.33 | 2.65 |
| 27 | *NnNDM3* | 104602643 / 37 | 0.90 | 1.65 | 0.42 |
| 28 | *NnDAHPS1* | 104604561 / 2 | 4.92 | 3.11 | 0.92 |
| 29 | *NnDAHPS2* | 104608661 / 77 | 1.20 | 0.41 | 1.48 |
| 30 | *NnDAHPS3* | 104606377 / 60 | 4.32 | 3.61 | 0.70 |
| 31 | *NnDHQS* | 104606748 / 62 | 1.40 | 1.27 | 1.01 |
| 32 | *NnDHD-SDH* | 104585714 / 3 | 1.05 | 0.72 | 3.91 |
| 33 | *NnSK* | 104600011 / 26 | 2.12 | 0.87 | 1.98 |
| 34 | *NnEPSPS* | 104602250 / 36 | 3.07 | 2.05 | 1.47 |
| 35 | *NnCS* | 104602632 / 37 | 1.50 | 0.99 | 1.53 |
| 36 | *NnCM1* | 104598462 / 1 | 2.42 | 1.88 | 0.64 |
| 37 | *NnCM2* | 104603298 / 41 | 2.10 | 1.83 | 0.96 |
| 38 | *NnPPA-AT* | 104600083 / 26 | 1.83 | 1.41 | 1.02 |
| 39 | *NnADH/PDH* | 104594520 / 11 | 26.57 | 4.22 | 2.59 |


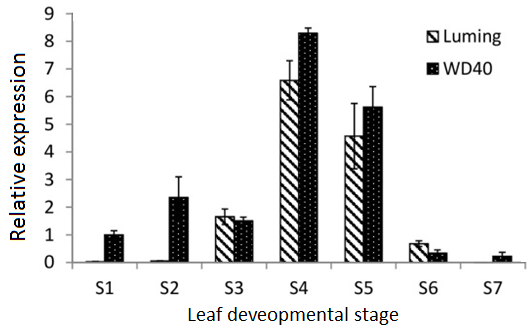


Fig. S1. The expression profile of the *NnNCS7* gene in leaf of two cultivars throughout the whole developmental stages.


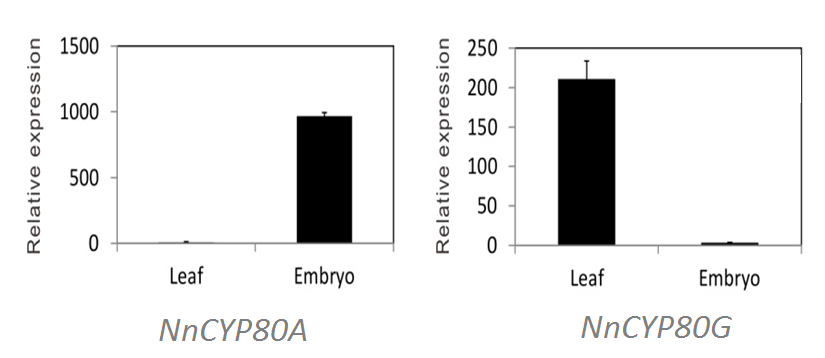


Fig. S2 qRT-PCR expression profiling of two *CYP80* genes in the leaf and embryo tissues of lotus.


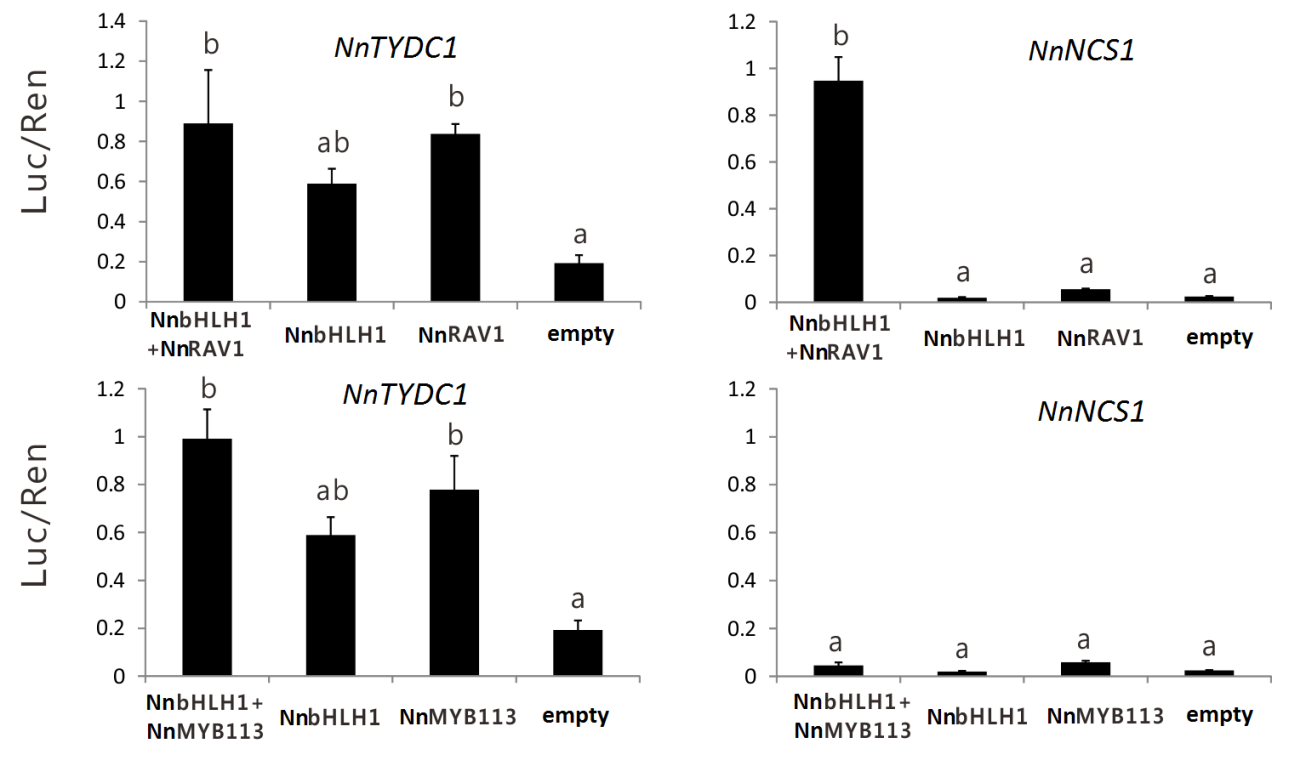


Figure S3 Estimation of effect of NnbHLH1 with NnMYB113 or NnRAV1 on activation of the promoter of BIA pathway genes using transient dual luciferase assay. Error bars show SE of three biological replicates. Different lowercase letters indicate significant difference at *P* < 0.05.
