# Supplementary material for: Investigation of benzylisoquinoline alkaloid biosynthetic pathway and its transcriptional regulation in lotus
Source: Hortic Res. 2018 Jun 1;5:29. doi: 10.1038/s41438-018-0035-0 (PMC5981371; doi:10.1038/s41438-018-0035-0)

**Supplementary data 1** Alignment of lotus BIA structural genes with reference genes from *Papaver somniferum* (Ps), *Eschscholzia californica* (Ec), *Coptis japonica* (Cj) *and Nicotiana tobacum* (Nt).

1. TYDC


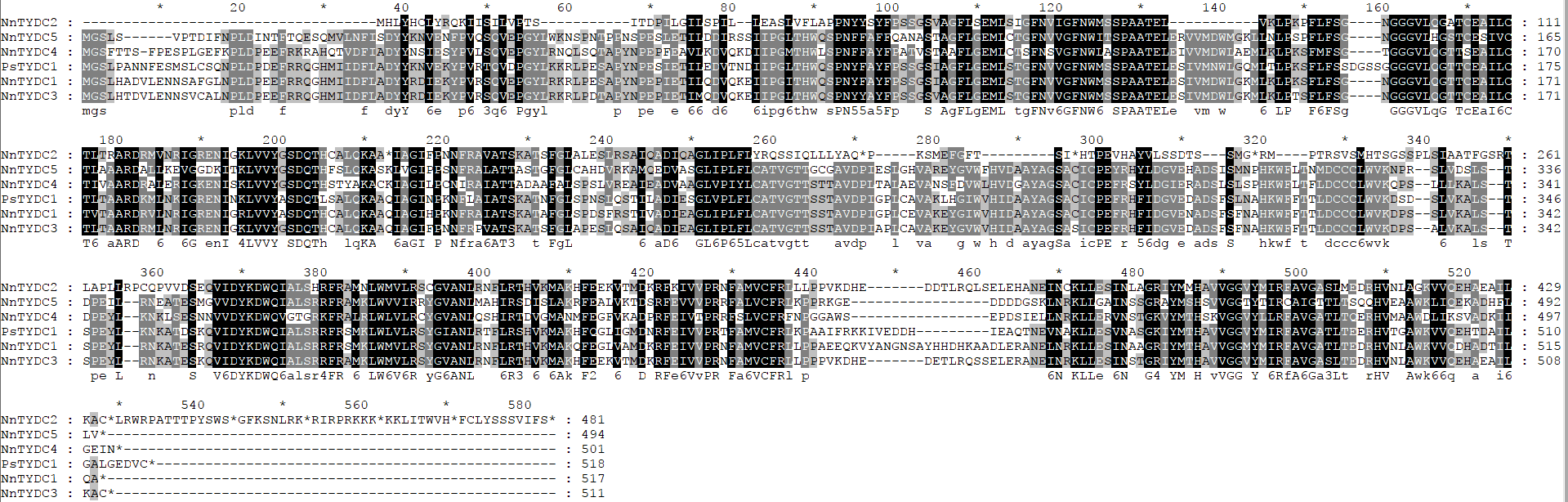


2. NCS


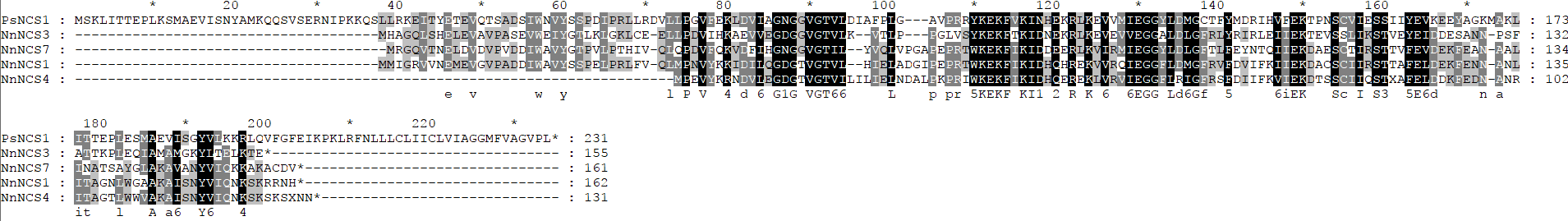


3. 6OMT/4’OMT


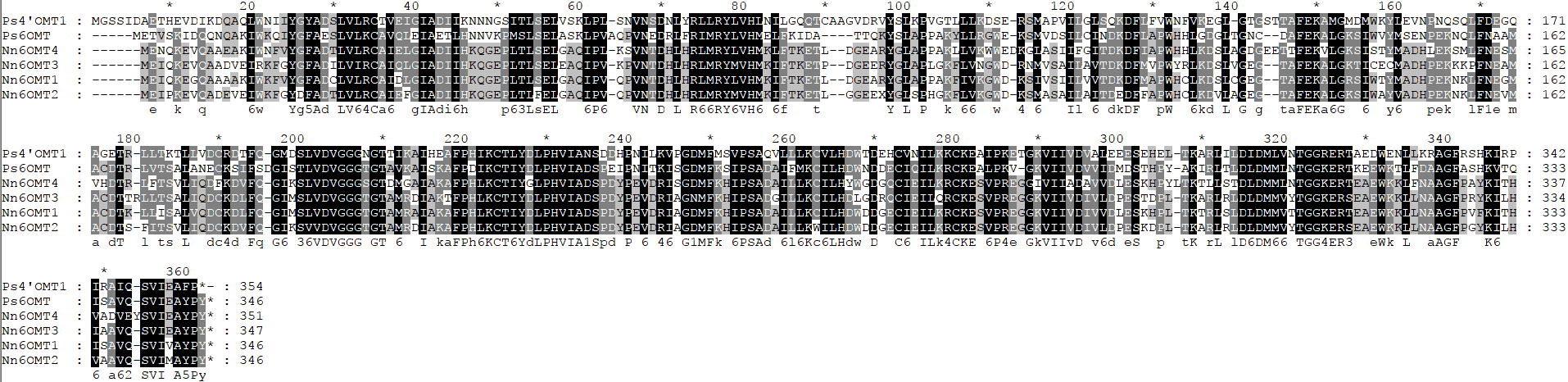


4. 7OMT


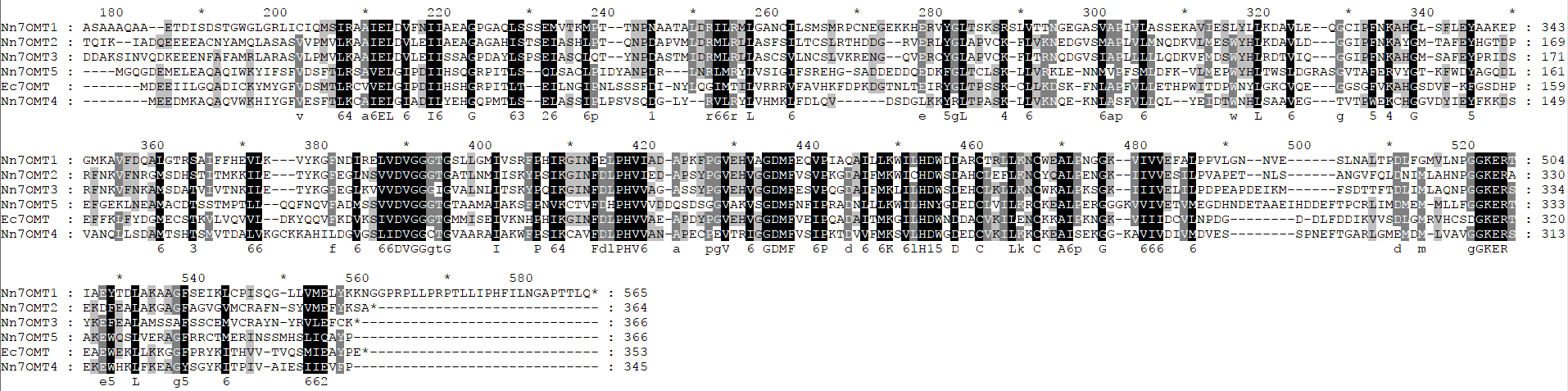


5. CYP80A/G


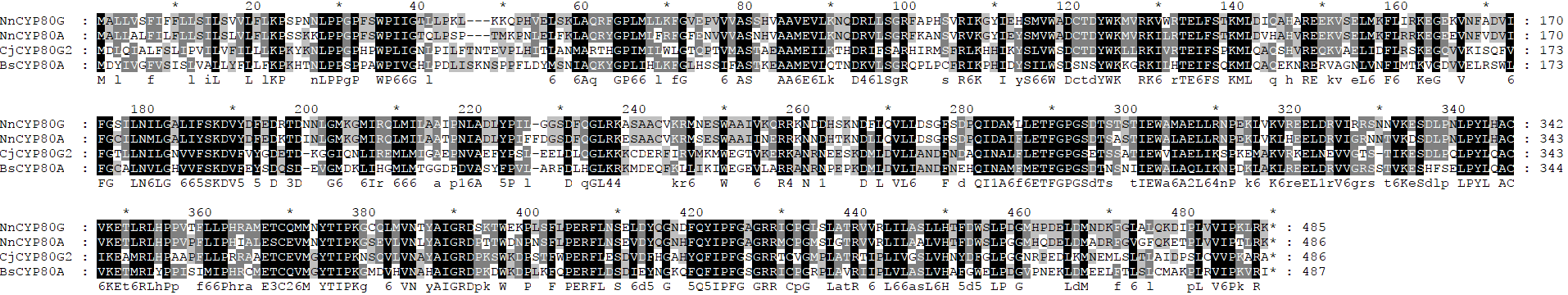


6. CYP719A


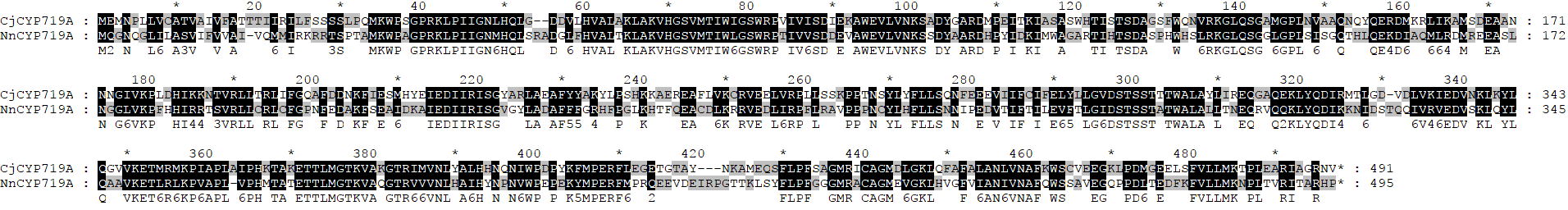


7. ODM


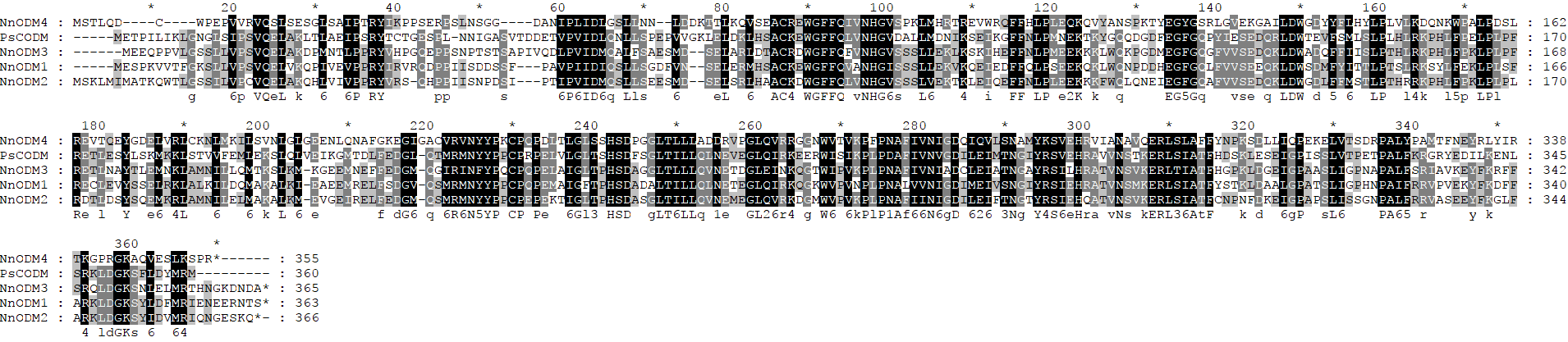


8. NDM


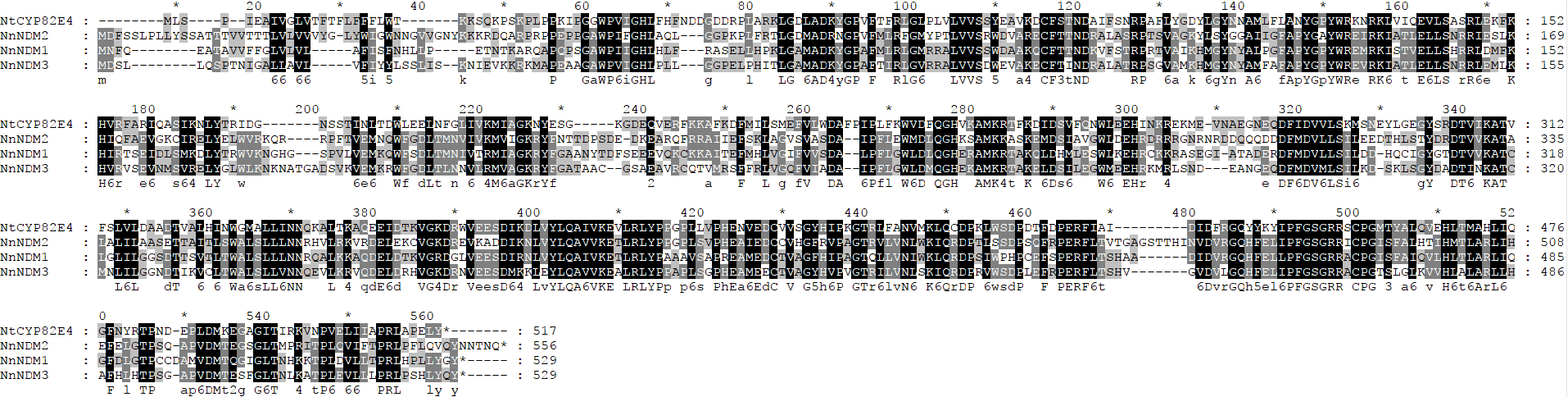

Supplement: Supplementary file 2 — Suppementary data 1 [file 41438_2018_35_MOESM2_ESM.docx]
